# Prostate MR image segmentation using a multi-stage network approach

**DOI:** 10.1007/s11255-025-04763-0

**Published:** 2025-09-05

**Authors:** Lars E. O. Jacobson, Mohamed Bader-El-Den, Lalit Maurya, Adrian A. Hopgood, Vincenzo Tamma, Shamsul K. Masum, David J. Prendergast, Peter Osborn

**Affiliations:** 1 School of Computing, University of Portsmouth, Portsmouth, UK; 2 Faculty of Technology, University of Portsmouth, Portsmouth, UK; 3 School of Mathematics and Physics, Institute of Cosmology and Gravitation, University of Portsmouth, Portsmouth, UK; 4 School of Electrical and Mechanical Engineering, University of Portsmouth, Portsmouth, UK; 5 Innovative Physics Ltd, Portsmouth, UK; 6 Portsmouth Hospitals, University NHS Trust, Portsmouth, UK

**Keywords:** Image segmentation, Prostate, Magnetic resonance imaging, U-net, End-to-end

## Abstract

Prostate cancer (PCa) remains one of the most prevalent cancers among men, with over 1.4 million new cases and 375,304 deaths reported globally in 2020. Current diagnostic approaches, such as prostate-specific antigen (PSA) testing and trans-rectal ultrasound (TRUS)-guided biopsies, are often Limited by low specificity and accuracy. This study addresses these Limitations by leveraging deep learning-based image segmentation techniques on a dataset comprising 61,119 T2-weighted MR images from 1151 patients to enhance PCa detection and characterisation. A multi-stage segmentation approach, including one-stage, sequential two-stage, and end-to-end two-stage methods, was evaluated using various deep learning architectures. The MultiResUNet model, integrated into a multi-stage segmentation framework, demonstrated significant improvements in delineating prostate boundaries. The study utilised a dataset of over 61,000 T2-weighted magnetic resonance (MR) images from more than 1100 patients, employing three distinct segmentation strategies: one-stage, sequential two-stage, and end-to-end two-stage methods. The end-to-end approach, leveraging shared feature representations, consistently outperformed other methods, underscoring its effectiveness in enhancing diagnostic accuracy. These findings highlight the potential of advanced deep learning architectures in streamlining prostate cancer detection and treatment planning. Future work will focus on further optimisation of the models and assessing their generalisability to diverse medical imaging contexts.

## Introduction

In 2020, the global incidence of prostate cancer (PCa) was estimated at 1,414,259 new cases, with mortality figures reaching 375,304 [1]. The prevalence of this Malignancy notably increases in men aged 65 and above. There is a pressing need for the development of non-invasive diagnostic modalities that can accurately differentiate the severity of PCa beyond the conventional approach of active surveillance [2]. The traditional diagnostic pathway for PCa hinges on the assessment of prostate-specific antigen (PSA) levels and the execution of trans-rectal ultrasound (TRUS)-guided biopsies. Despite its widespread use, PSA screening is plagued by a low specificity rate of approximately 36%, due to the elevation of PSA levels in benign prostatic conditions [3]. This diagnostic ambiguity highlights the inherent Limitations of PSA as a reliable biomarker, where elevated levels do not conclusively indicate the presence of a tumour, nor do normal levels definitively exclude it. Moreover, the TRUS-guided biopsy, which predominantly targets the peripheral aspects of the gland, suffers from methodological drawbacks. Given that 30–40% of PCa originate in the anterior midline transition zone (TZ), a significant proportion of tumours may elude detection with this approach due to the systematic but random sampling of the peripheral zone (PZ), compounded by ultrasound’s poor capability in distinguishing cancerous tissues from benign ones [4].

Against this backdrop, magnetic resonance (MR) imaging emerges as an alternative, offering the potential to significantly enhance diagnostic accuracy. MR imaging has advanced capabilities in tumour detection both pre- and post-biopsy and sets the stage for a shift in PCa diagnostics. The high-resolution imaging and detailed tissue characterisation afforded by MR imaging not only facilitate the precise localisation of tumours within the prostate gland but also aid in the assessment of tumour aggressiveness. This technological advancement underscores the imperative for integrating MR imaging into the diagnostic workflow, promising a leap towards more accurate, timely, and non-invasive detection of PCa, thereby addressing the critical limitations of current diagnostic practices[5–8].

The Prostate Imaging Reporting and Data System (PI-RADS) represents a significant advancement in the domain of PCa diagnostics, providing a standardised framework for the evaluation and characterisation of prostate tumours via MR imaging. This scoring system, ranging from 1 to 5, facilitates the stratification of cancer risk based on MRI findings. Specifically, the PI-RADS methodology involves the delineation of the prostate into its two main anatomical zones—the transition zone (TZ) and the peripheral zone (PZ)—as identified on MR images.

**Fig. 1:**
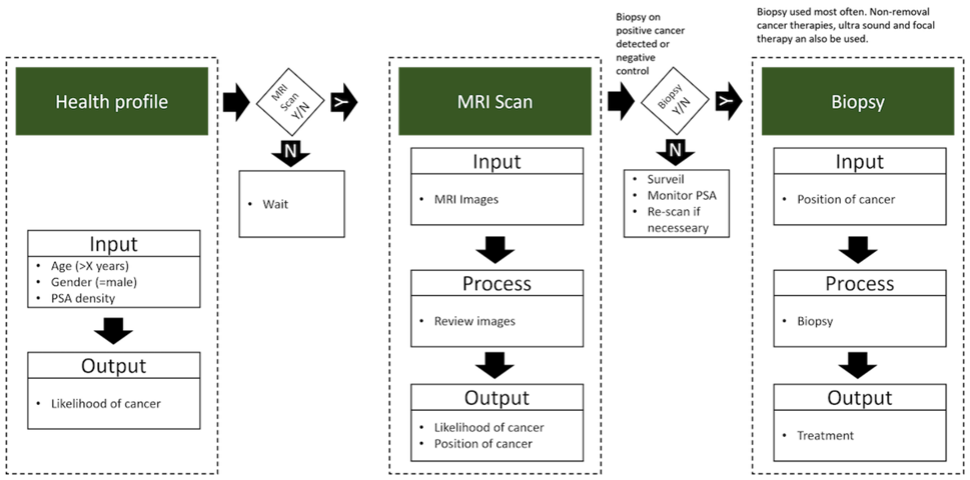
Process description of prostate cancer (PCa) procedure and diagnosis. The health profile of the patient is the initial input where properties e.g. age and PSA density are evaluated to determine if the patient should progress in the process. A magnetic resonance (MR) scan is executed and from this the likelihood and position of cancer is retrieved. For the final step, a biopsy is performed with input from the previous steps [9]

This paper presents several contributions to the field of prostate MR image segmentation. First, it introduces a systematic approach to annotation, where the prostate’s transition zone (TZ) and peripheral zone (PZ) are clearly delineated using expert-reviewed annotations. Second, a robust and reproducible data preparation pipeline was implemented to ensure consistent model training across CNN architectures. While the individual preprocessing techniques (e.g., normalisation, augmentation) are widely used in the field, we provide a systematic and well-documented pipeline optimised for multi-stage segmentation evaluation. Finally, this paper contributes by systematically evaluating a range of established CNN architectures within a controlled framework, comparing their performance across different segmentation strategies—namely, one-stage, sequential two-stage, and end-to-end two-stage approaches. This evaluation provides new insights into how architectural choices and segmentation design impact performance in multi-zone prostate MR segmentation. While several publicly available datasets provide valuable resources for prostate cancer assessment, this work specifically focuses on expert-reviewed annotations of both the transition zone (TZ) and peripheral zone (PZ) across all axial MR slices. Unlike lesion detection datasets, the dataset used in this paper is optimised for zone-level segmentation tasks and enables structured evaluation of multi-stage segmentation pipelines. The annotation process was iteratively validated and standardised to support training of consistent and generalisable CNN models (Fig. 1).

## Related work

To achieve automated prostate segmentation, many methods and algorithms have been proposed ranging from traditional machine learning techniques to convolutional neural networks (CNNs) [10]. CNN-based methods have gained prominence for their hierarchical feature extraction and robustness in medical image analysis [11].

Multiple studies have investigated CNN approaches for prostate segmentation. Li et al. used a Dense U-Net in a two-stage pipeline for improved segmentation accuracy [11]. Ing et al. leveraged SegNet variants for prostate zone classification [12], and Erkus et al. demonstrated that DeepLabV3 outperformed traditional U-Net in MR image segmentation tasks [13]. Wang et al. proposed a two-stage approach with a squeeze-excitation module and RAUNet to segment prostate regions effectively [14]. Bouslimi et al. achieved high accuracy using MultiResUNet and augmented data [15], while Li et al. addressed blurred features via a pyramid pool U-Net [16]. Ding et al. incorporated attention modules in U-Net to recalibrate multi-level feature maps [17].

Litjens et al. [18] demonstrated the use of MR image segmentation in combination with PI-RADS scoring for automated PCa diagnosis. Bardis et al. confirmed that multi-U-Net architectures could match radiologist-level performance, and Luo et al. [7] showed that preprocessing using RLRE algorithms can enhance MRI quality.

End-to-end models have emerged as strong contenders for segmentation. Wang et al.’s RAUNet model achieved a Dice Similarity Coefficient (DSC) of 0.904. Tian et al. [19] combined ConvLSTM and Gated Graph Neural Networks for accurate 3D prostate segmentation. The nnU-Net framework [20] demonstrated high adaptability and generalisation by auto-configuring its pipeline based on the input dataset.

Lastly, ensemble learning techniques have also been used to improve segmentation outcomes, as shown by Litjens et al. [21], who used majority voting across multiple models to boost performance and efficiency in clinical applications.

Recent advances in other domains have also demonstrated the value of hybrid architectures and advanced feature extraction. Transformer-based models have emerged as state-of-the-art in medical image segmentation. TransUNet [22] combines a Vision Transformer (ViT) encoder with a U-Net decoder, achieving strong performance in abdominal organ segmentation. UNETR [23] and Swin-UNet [24] have also demonstrated high accuracy and better long-range dependency modeling, which is particularly valuable in volumetric medical data. Although not yet widely applied to prostate datasets, transformer-based architectures have demonstrated strong performance in complex segmentation tasks such as brain tumor (BraTS) and multi-organ CT segmentation. Their ability to model long-range dependencies through self-attention mechanisms presents a valuable opportunity for improving prostate zone delineation, which often suffers from ambiguous boundaries. Cross-domain research has also highlighted the value of robust feature representation. For instance, continuous wavelet transforms and spatio-temporal attention modules have enhanced fault classification in industrial systems by extracting multiscale features from non-stationary signals [25]. Similarly, the integration of S-Transform scalograms with CNN-KAN architectures has improved classification accuracy in centrifugal pump monitoring [26]. Further, Siddique et al. [27] recently proposed a dual-input convolutional neural network (CNN) that fuses acoustic emission and vibration signals to enhance fault classification in mechanical systems. This multimodal approach highlights the benefits of complementary feature streams and cross-domain sensor fusion in improving model robustness and discriminative capacity. While developed in a different context, the underlying principle—leveraging diverse information sources to enrich representation learning—bears relevance for future prostate segmentation research. Specifically, integrating multi-parametric MR sequences or combining spatial and temporal priors could yield analogous performance gains in anatomically complex or low-contrast prostate regions. These approaches address challenges analogous to those found in prostate MRI segmentation—namely, the need to detect subtle structural variations under noisy or complex signal conditions. The principles of hierarchical feature fusion, temporal-frequency encoding, and hybrid attention-based learning could be repurposed to improve anatomical boundary detection in MRI, particularly in regions with low tissue contrast, such as the prostate base and apex.

## Methods

**Fig. 2:**
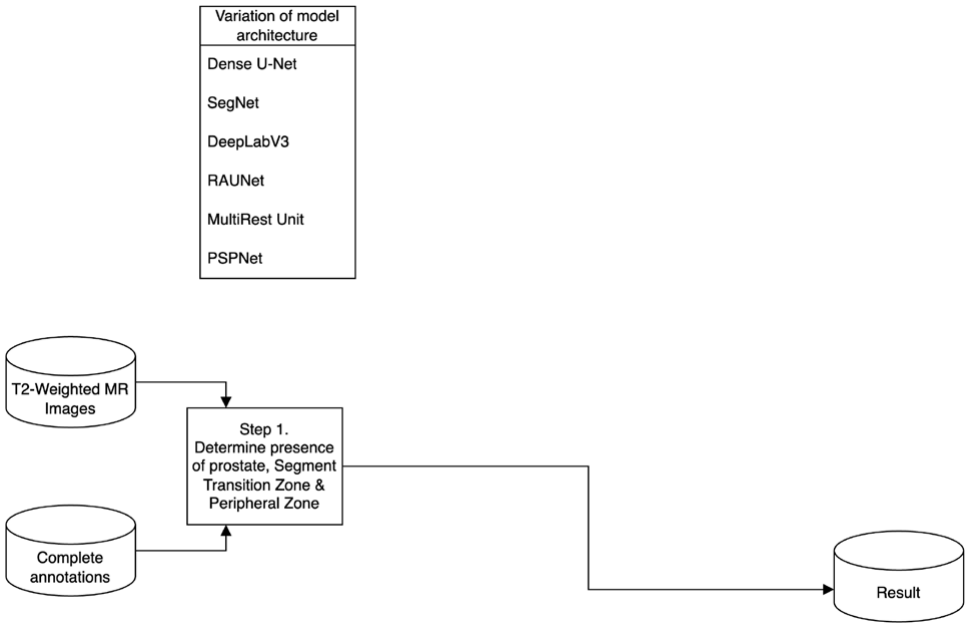
One-stage segmentation workflow: this approach utilises T2-weighted MR images with complete annotations for direct segmentation of the prostate, focusing on the transition zone (TZ) and peripheral zone (PZ). Various model architectures such as Dense U-Net, SegNet, DeepLabV3, RAUNet, MultiResUNet, and PSPNet were tested to evaluate their efficacy in a single-step segmentation process

**Fig. 3:**
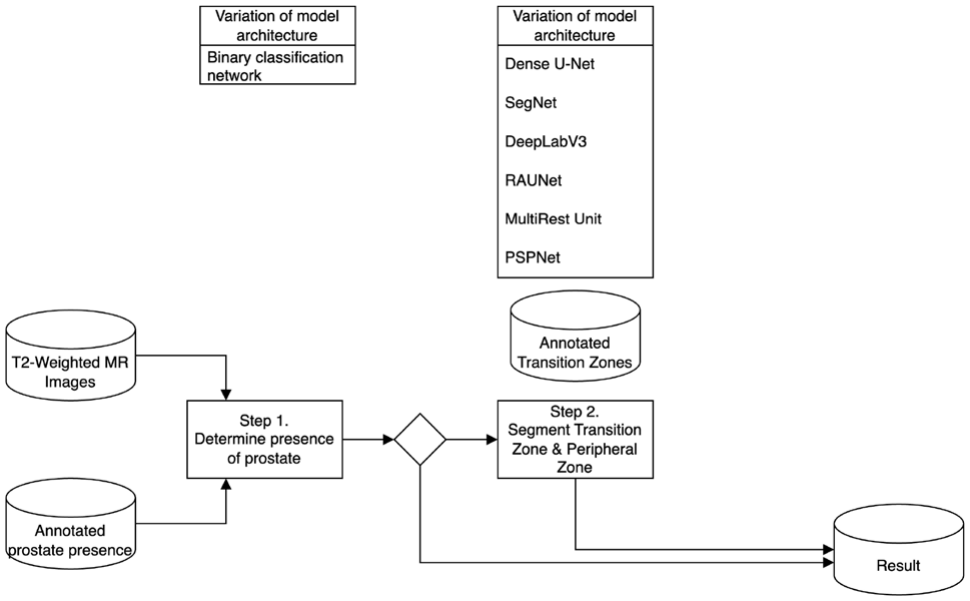
Sequential two-stage prostate segmentation network: this workflow implements a sequential approach where the first stage involves a binary classification network to determine prostate presence in T2-weighted MR images. If the prostate is detected, the process moves to the second stage, which uses one of the model architectures (Dense U-Net, SegNet, DeepLabV3, RAUNet, MultiResUNet, PSPNet) to perform segmentation of the transition zone (TZ) and peripheral zone (PZ)

**Fig. 4:**
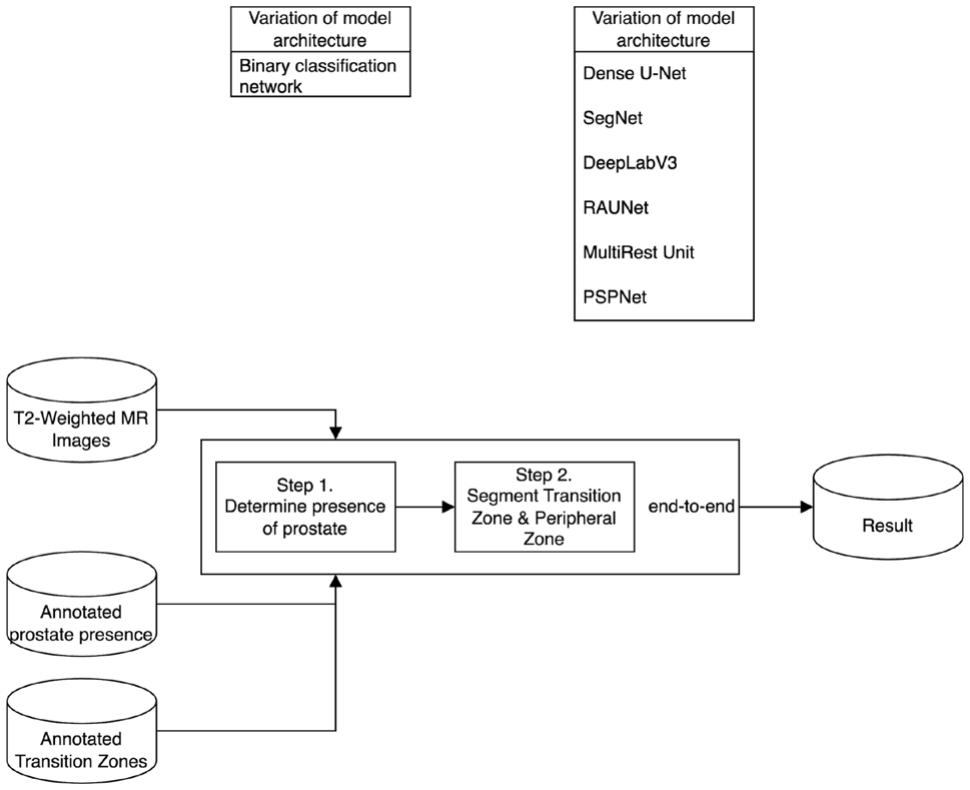
End-to-end two-stage prostate segmentation network: this workflow integrates an end-to-end approach where the binary classification and segmentation tasks are combined into a single pipeline. Upon detection of the prostate, the model simultaneously segments the transition zone (TZ) and peripheral zone (PZ) using the selected architecture (Dense U-Net, SegNet, DeepLabV3, RAUNet, MultiResUNet, PSPNet)

In this study, multiple model architectures were employed to evaluate their effectiveness in prostate MR image segmentation through a multi-stage approach. Three distinct segmentation strategies were proposed to comprehensively assess model performance: a one-stage approach, a sequential two-stage approach, and an end-to-end two-stage approach. These approaches are illustrated in Figs. 2, 3 and 4, respectively.

In the one-stage approach, the model directly performs prostate segmentation from the T2-weighted MR images. This method allows for a comprehensive analysis of how different architectures manage segmentation tasks without the preliminary step of prostate detection.

The Sequential Two-stage approach introduces a binary classification step to detect the presence of the prostate before proceeding to the segmentation task. This strategy aims to filter out irrelevant images, thereby improving segmentation efficiency and potentially enhancing overall model performance.

The end-to-end two-stage approach combines the detection and segmentation stages into a single cohesive pipeline, facilitating simultaneous learning and prediction, which can potentially enhance the accuracy and computational efficiency of the segmentation process.

The proposed methods and algorithm flow encompass the following core components:

- Pre-processing of T2-weighted MR images, including normalisation to ensure image quality consistency.
- Segmentation of the prostate into the transition zone (TZ) and peripheral zone (PZ) based on single-class annotations.

**Fig. 5:**
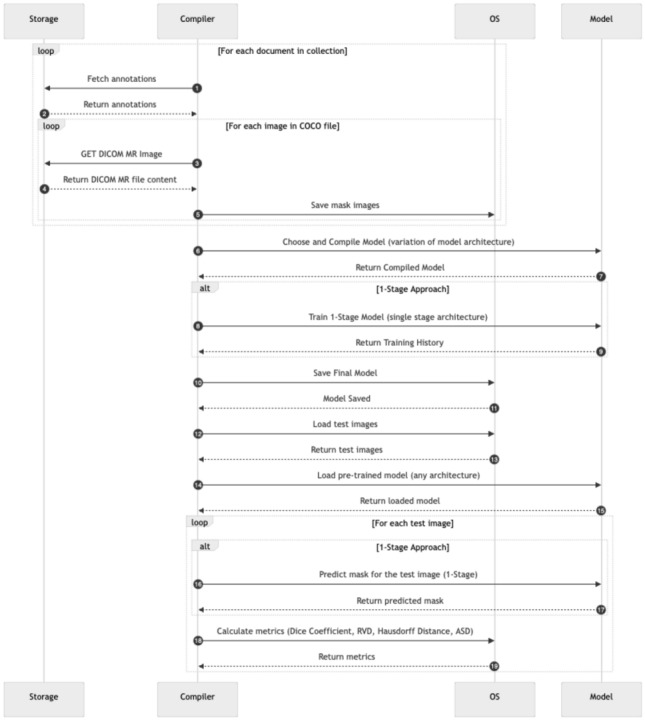
One-stage model training and testing workflow: this diagram illustrates the sequential steps of processing annotations and DICOM MR images to train the model using the one-stage approach. The workflow includes compiling the model with the selected architecture, training, and evaluation using metrics such as Dice Coefficient, Relative Volume Difference (RVD), Hausdorff Distance, and Average Surface Distance (ASD)

**Fig. 6:**
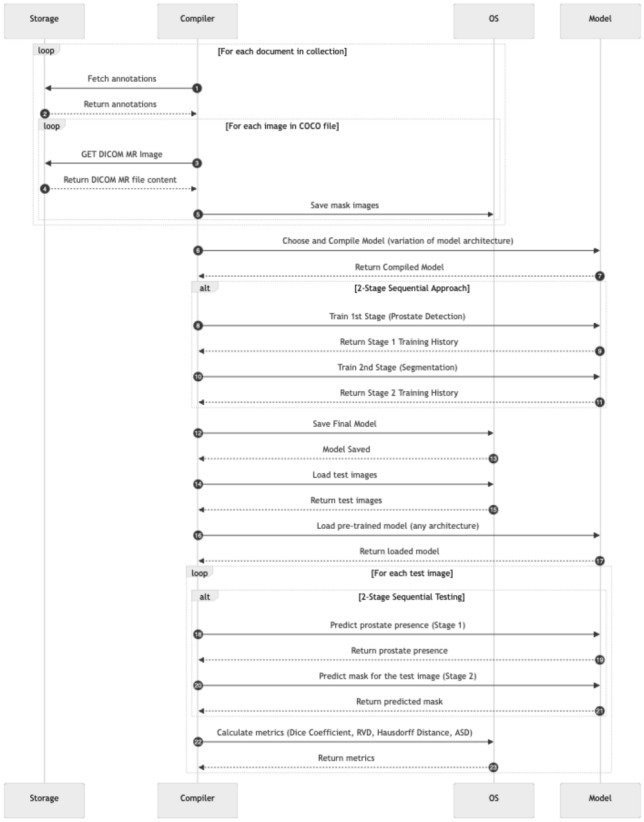
Sequential two-stage model training and testing workflow: this diagram shows the training process for the two-stage sequential approach, highlighting the binary classification in stage 1 followed by the segmentation in stage 2. Performance metrics are calculated for each test image to evaluate the effectiveness of the approach

**Fig. 7:**
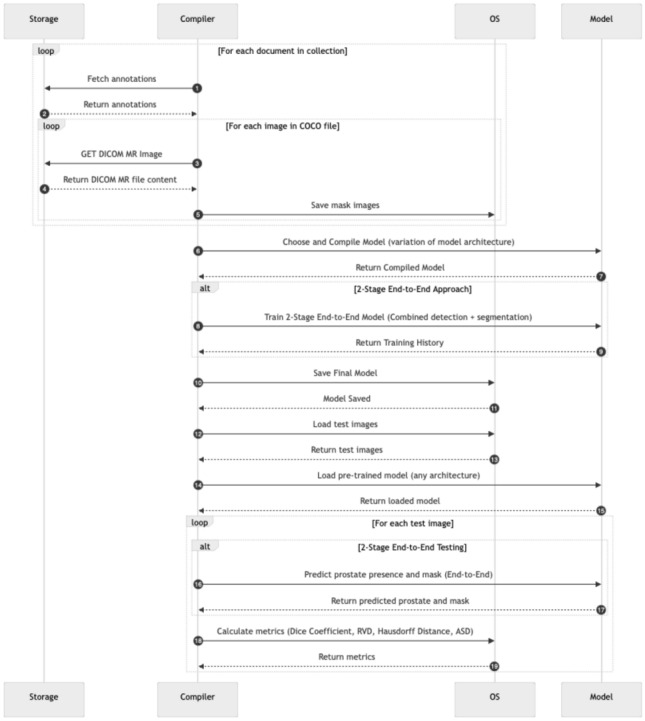
End-to-end Two-stage model training and testing workflow: This diagram presents the end-to-end training pipeline, which combines prostate detection and segmentation into a unified model. The model is evaluated using performance metrics calculated in a single pass for each test image

The execution process for each approach is depicted in Figs. 5, 6 and 7. This process starts with the aggregation of data from the centralised repository of annotations, where T2-weighted MR images, along with their corresponding annotations enriched with metadata (e.g., Patient ID, expert annotations), are collated. The raw MR images and their annotations undergo pre-processing involving intensity normalisation, data augmentation techniques to enhance diversity, and conversion from DICOM to more universally compatible formats. This pre-processing stage ensures that the dataset is suitable for training the proposed models. While the preprocessing steps used in this study follow established practices in medical image analysis, the key contribution lies in their tailored integration to support the training of one-stage and two-stage segmentation pipelines under consistent experimental settings.

By incorporating predicted segmentation masks back into the training dataset, validated through expert opinion, the model undergoes iterative refinement to enhance segmentation accuracy.

### Patient and public involvement

It was determined not possible to involve patients or the public in the design of this research. This research aims to produce generalisable results using data from The Cancer Imaging Archive (TCIA), which is publicly available [28]. The data set used for PCa consists of 61,119 t2-weighted MR images for 1151 patients.

### Model architectures

This research implements six deep learning architectures for prostate MR image segmentation: Dense U-Net, SegNet, DeepLabV3, RAUNet, MultiResUnet, and PSPNet. The Attention U-Net is implemented by incorporating attention gates into the skip connections, modulating feature maps between the encoder and decoder. DeepLabV3+ utilises Atrous Spatial Pyramid Pooling (ASPP) with different dilation rates and a backbone of Xception, followed by upsampling to restore original image resolution. DenseUNet is implemented with dense blocks that connect each layer to all previous layers, with upsampling handled through concatenation layers. MultiResUNet includes multi-resolution blocks and residual connections, facilitating feature extraction across different scales. RAUNet combines residual blocks with attention mechanisms, where attention gates are added to the skip connections. SegNet uses an encoder-decoder structure with pooling and unpooling layers to restore spatial resolution, and the output is generated through softmax layers. PSPNet is implemented with a pyramid pooling module that aggregates features at multiple scales, followed by upsampling and concatenation to integrate local and global context. While transformer-based or hybrid models (e.g., TransUNet, Swin-UNet) have demonstrated strong performance in recent segmentation studies, this work focuses exclusively on CNN-based architectures. This choice was made to maintain architectural consistency and allow for a controlled comparison across one-stage, sequential two-stage, and end-to-end two-stage strategies. CNNs also remain computationally efficient and are widely adopted in clinical and embedded pipelines, making this study relevant for practical deployment.

#### Dense U-Net

The Dense U-Net architecture incorporates densely connected convolutional blocks, where each layer is connected to every other layer in a feed-forward Manner to ensure Maximum information flow. The growth rate was set to 32, and 4 layers per dense block were used. The model employed batch normalisation after every convolutional layer, and the Adam optimiser with an initial learning rate of $1e-4$ was utilised. Dropout was applied with a rate of 0.2 to prevent overfitting.

$$f(x) = W \cdot x + b$$

where $f(x)$ represents the dense layer’s output, $W$ are the weights, and $b$ is the bias.

#### SegNet

The SegNet architecture was configured with an encoder-decoder structure. The encoder utilised pre-trained weights from VGG16, and the decoder mirrored the encoder structure using upsampling layers. ReLU activation functions were applied after every convolutional layer, and batch normalisation was used. The model was trained using the categorical cross-entropy loss function with an Adam optimiser at a learning rate of $1e-3$. The number of filters in each layer was adjusted to match the input resolution (128 $\times$ 128 pixels).

#### DeepLabV3

The DeepLabV3 model was implemented using the Xception backbone pre-trained on ImageNet. Atrous Spatial Pyramid Pooling (ASPP) with dilation rates set to 6, 12, and 18 was incorporated to capture multi-scale information. The output from the ASPP module was concatenated and passed through two convolutional layers. The model was optimised using the Adam optimiser with a learning rate of $1e-4$, and a combination of Dice loss and categorical cross-entropy was employed to address class imbalance.

$$ASPP(x) = \sum _{i=1}^{N} W_i *x$$

where $W_i$ denotes convolution filters, $*$ represents the atrous convolution operation, and $N$ is the number of filters.

#### RAUNet (residual attention U-Net)

The RAUNet model combines residual blocks with attention gates to refine feature Maps. The residual blocks were implemented with skip connections, and attention blocks were incorporated to emphasise relevant regions. The number of filters in each block was set to 64, 128, 256, and 512 for the successive layers. The model was trained using the Adam optimiser with an initial learning rate of $1e-4$, and a combined loss of Dice and categorical cross-entropy was applied to handle imbalanced classes. Batch normalisation and ReLU activation were utilised after every convolutional layer.

#### MultiResUnet

The MultiResUnet architecture was implemented with multi-resolution blocks to capture features at various scales. Each multi-resolution block combined convolution operations with different kernel sizes (3 $\times$ 3, 5 $\times$ 5, and 7 $\times$ 7). A residual path was added to each block to facilitate gradient flow. The alpha value was set to 1.67, following the original configuration, and batch normalisation was applied after every convolution. The optimiser used was Adam, with an initial learning rate of $1e-4$. The loss function was configured as a combination of Dice loss and categorical cross-entropy to address class imbalance.

#### PSPNet (pyramid scene parsing network)

The PSPNet model incorporated pyramid pooling modules with bin sizes of [1, 2, 3, 6], enabling the model to capture global contextual information by pooling features at different scales. The output was concatenated and passed through convolutional layers with 256 filters. The model was trained using the Adam optimiser, with an initial learning rate of $1e-3$, and utilised a combined loss function of Dice and categorical cross-entropy to enhance performance.

$$y = W_1 \cdot f(x) + W_2 \cdot g(x)$$

where $y$ denotes the output feature map, $W_1$ and $W_2$ are weights, and $f(x)$, $g(x)$ represent feature maps from different scales.

### Data description and preparations

The data relate to PCa medical applications and have been sourced from The Cancer Imaging Archive (TCIA), which is publicly available [28]. The data set used for PCa consists of 61,119 t2-weighted MR images for 1,151 patients.

**Fig. 8:**
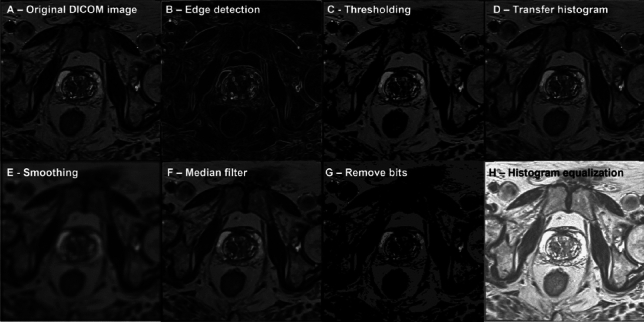
Pre-processing of one MR image. The original images undergo several pre-processing procedures i.e. B-Edge detection, C-Thresholding, D-Transfer histogram, E-Smoothing, F Median filter, G Remove bits and H-Histogram equalisation

The conversion of original images from the Digital Imaging and Communications in Medicine (DICOM) format to Portable Network Graphics (PNG) is a critical preprocessing step to facilitate the segmentation and analysis within this research framework. DICOM, being the standard for storing and transmitting information in medical imaging, encapsulates not only the image data but also a rich set of metadata, including patient information, imaging parameters, and device details. However, for the purposes of segmentation and analysis using deep learning models such as U-net, it is imperative to work with a more universally accessible and computationally manageable image format like PNG. The conversion process involves extracting the image data from the DICOM files and then standardising this data into the PNG format, which is widely supported by image processing libraries and tools. This transformation enables a streamlined workflow for image segmentation tasks, ensuring compatibility across various computing platforms and software environments. Additionally, converting to PNG may also involve adjustments in image contrast and brightness to enhance feature visibility for more effective segmentation and analysis, thereby optimising the input for the AI models employed in the study (Fig. 8).

For preprocessing, images and masks are first loaded and normalised. Images are resized to $128 \times 128$ pixels and converted to RGB format if necessary, ensuring uniform input size for all models. Masks are processed by converting colour values to class labels, followed by one-hot encoding into four classes. A custom data generator is implemented to handle imbalanced data, incorporating class weights that are computed from the training mask distribution. The data generator is also applying sample-wise weights during training, ensuring that each class is represented correctly in the loss calculations.

For all architectures, data augmentation techniques, including rotation, translation, and flipping, were applied to improve model generalisation. A batch size of 16 was used, and training was conducted for 50 epochs with early stopping based on validation loss. Learning rate reduction on plateau was implemented to adaptively reduce the learning rate when the validation performance plateaued.

The combination of Dice loss $L_{Dice}$ and categorical cross-entropy $L_{CCE}$ was employed as the primary loss function for all architectures:

$$L_{total} = \alpha L_{Dice} + \beta L_{CCE}$$

where $\alpha = 0.5$ and $\beta = 0.5$ to balance the contributions of both loss components.

For testing, the model is loaded with custom objects, including the combined loss and Dice similarity coefficient. Test images are read and preprocessed by resizing them to $128 \times 128$ and normalising pixel values. If the images are greyscale or contain an alpha channel, they are converted to RGB format and expanded to include a batch dimension. The model then predicts the segmentation mask for each test image, and the predicted class labels are mapped to colour-coded segmentation masks using a predefined colour scheme. To evaluate segmentation performance, ground truth masks are loaded, resized, and converted from one-hot encoding to class labels for comparison with the predicted masks.

Data augmentation is employed, applying transformations such as rotation, width/height shift, zoom, and horizontal flipping. This is applied to both the original images and annotations to prevent over-fitting and enhance generalisation. The augmented images and annotations are then passed through a custom generator that computes sample weights based on class distributions and yields batches of images, masks, and sample weights for training.

### External validation using the PI-CAI dataset

To assess the generalisability of the proposed segmentation framework, we conducted an external validation experiment using data from the PI-CAI (Prostate Cancer AI) challenge dataset [29]. This dataset comprises a large, multi-institutional collection of prostate MRI scans, annotated and curated for benchmarking AI models. For this experiment, we selected a subset of 100 patients from the publicly released T2-weighted series, for which anatomical delineations of the peripheral zone (PZ) and transition zone (TZ) are available via the anatomical annotations provided by Yuan et al. [30].

The best-performing model from this study was applied directly to the external dataset without retraining. Pre-processing steps included resizing all images to $128 \times 128$ pixels, normalisation, and RGB conversion to maintain consistency with the original training pipeline. Ground truth masks were generated from the zonal annotations following the same colour mapping and one-hot encoding scheme as used for the internal dataset. All segmentation predictions were evaluated using the Dice Similarity Coefficient (DSC) and Hausdorff Distance (HD) metrics. Relative Volume Difference (RVD) and Average Surface Distance (ASD) were excluded due to inconsistencies in mask formatting across the PI-CAI annotations. These results are reported separately in the Results section to demonstrate cross-dataset performance and further validate the robustness of this study’s proposed framework.

### Calculation of accuracy, sensitivity, and specificity

The evaluation metrics, namely accuracy, sensitivity, and specificity, were calculated to assess the model’s performance in both binary classification (prostate presence detection) and multi-class segmentation (Transition Zone - TZ, Peripheral Zone - PZ). These metrics provide insight into the model’s ability to correctly classify and segment prostate zones, which is critical for accurate diagnosis and treatment planning.

*a) Binary Classification (Prostate Presence)* For the binary classification task, accuracy, sensitivity, and specificity were computed using the following formulas:

1 $$\begin{aligned} & \text {Accuracy} = \frac{TP + TN}{TP + TN + FP + FN} \end{aligned}$$

2 $$\begin{aligned} & \text {Sensitivity} = \frac{TP}{TP + FN} \end{aligned}$$

3 $$\begin{aligned} & \text {Specificity} = \frac{TN}{TN + FP} \end{aligned}$$

where:

- $TP$: True Positives (prostate presence correctly identified),
- $TN$: True Negatives (correctly identified as absence of prostate),
- $FP$: False Positives (incorrectly identified as prostate presence),
- $FN$: False Negatives (missed prostate presence).

*b) Multi-Class Segmentation (Prostate Zones)* For the segmentation of prostate zones, each class (background, TZ, and PZ) was treated independently. For each class, sensitivity and specificity were calculated by converting the problem to a binary classification (presence vs. absence of the class in each pixel). The calculations used were:

4 $$\begin{aligned} & \text {Sensitivity (per class)} = \frac{TP_{\text {class}}}{TP_{\text {class}} + FN_{\text {class}}} \end{aligned}$$

5 $$\begin{aligned} & \text {Specificity (per class)} = \frac{TN_{\text {class}}}{TN_{\text {class}} + FP_{\text {class}}} \end{aligned}$$

where $TP_{\text {class}}, TN_{\text {class}}, FP_{\text {class}},$ and $FN_{\text {class}}$ refer to true positives, true negatives, false positives, and false negatives for each specific class. The final accuracy for segmentation was averaged over all pixels in each class.

### Evaluation metrics for prostate segmentation model

In evaluating the performance of the medical imaging prostate segmentation model, several metrics are employed to assess the accuracy and robustness of the segmentation results. For models that use a two-stage approach, metrics are calculated separately for each stage before combining them to evaluate the overall performance. The first stage involves detecting the presence of the prostate, and its performance is evaluated using binary classification metrics, specifically sensitivity, specificity, and accuracy, to assess how well it distinguishes prostate presence from absence.

The second stage then performs segmentation on images where the prostate is detected. Here, segmentation-specific metrics, including the Dice Similarity Coefficient (DSC), Relative Volume Difference (RVD), Hausdorff Distance (HD), and Average Surface Distance (ASD), are used to measure the accuracy of boundary delineation for different prostate zones (e.g., Transition Zone and Peripheral Zone). The DSC quantifies the overlap between the predicted and ground truth segmentations, calculated as:

$$DSC = \frac{{2 \times TP}}{{2 \times TP + FP + FN}}$$

where $TP$ represents true positives, $FP$ false positives, and $FN$ false negatives. This metric assesses the degree of overlap, with higher DSC values indicating better segmentation accuracy.

The Relative Volume Difference (RVD) measures the discrepancy in volume between the predicted and ground truth segmentations, expressed as:

$$RVD = \frac{{V_p - V_{gt}}}{{V_{gt}}}$$

where $V_p$ and $V_{gt}$ denote the volumes of the predicted and ground truth segmentations, respectively. RVD evaluates how closely the predicted segmentation volume matches the ground truth.

The Hausdorff Distance (HD) captures the maximum distance between corresponding points in the predicted and ground truth segmentations, offering insight into the worst-case boundary discrepancies. The Average Surface Distance (ASD) computes the average distance between the surfaces of the two segmentations, providing a more generalised assessment of boundary alignment.

For the two-stage approach, the final accuracy of the entire workflow is calculated as the average of the classification accuracy (A1) from Stage 1 and the segmentation accuracy (A2) from Stage 2. In some cases, a weighted approach is used, giving equal importance to both stages, or alternatively, an overall workflow accuracy is computed by multiplying the classification and segmentation accuracies. This multi-stage evaluation strategy ensures a comprehensive assessment of both the model’s ability to detect prostate presence and its precision in delineating prostate boundaries. These metrics collectively provide a thorough evaluation of the model’s effectiveness in accurately identifying and segmenting prostate boundaries in medical images [31].

### Annotations and training

**Fig. 9:**
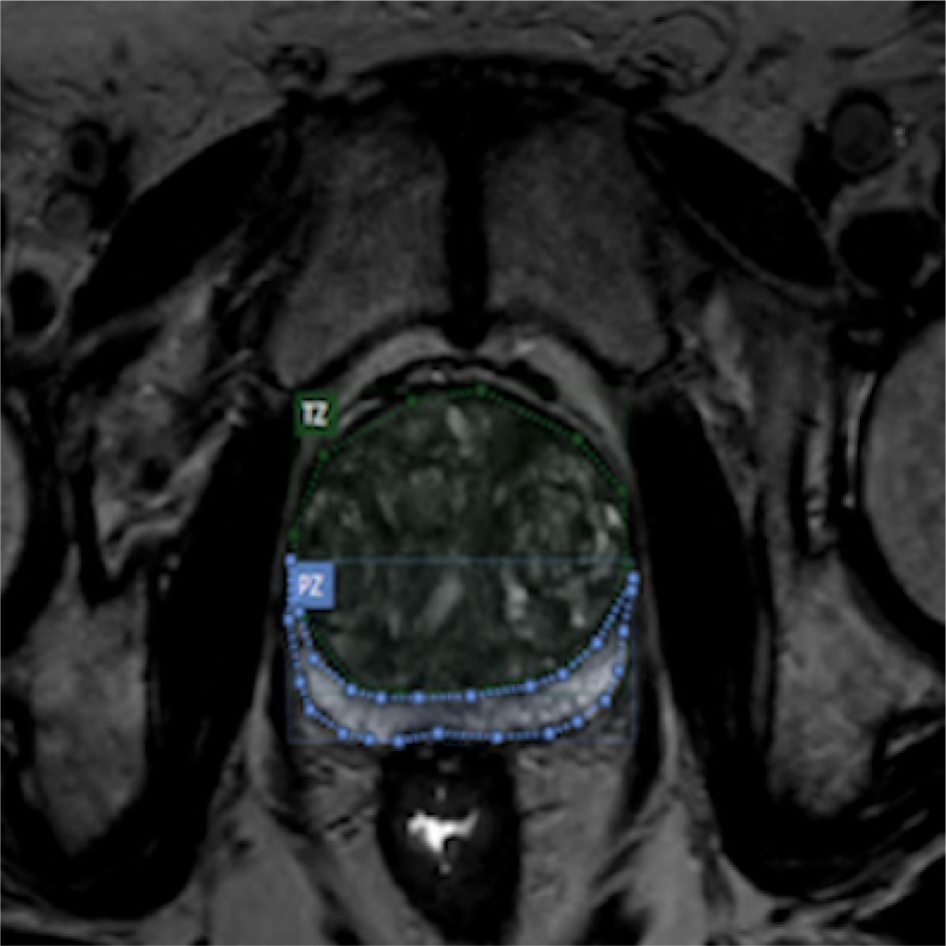
Transition zone (TZ) and peripheral zone (PZ) annotated combined with the original image

The MR images used are not pre-annotated with TZ or PZ. Figure 9 displays the TZ and PZ on an MR image. An iterative approach has been used, where the annotations are perfected over time. To ensure anatomical accuracy of TZ and PZ delineations, we employed an iterative annotation refinement process involving two expert radiologists. Initial annotations were reviewed in multiple rounds, with feedback integrated into successive versions. Although we did not explicitly quantify inter-observer variability in this study, care was taken to standardize boundary decisions—especially in ambiguous cases such as anterior TZ blending into the peripheral zone. Each recurrence of any of the zones is required to perform PI-RADS scoring [32, 33] and requires each MR image per patient to be labelled appropriately. Typically, in TCIA [28] data sets, each patient has a collection of MR images with a median of 60 slices or images. Additionally, each patient has been evaluated using the UCLA (University of California, Los Angeles) PCa index following PI-RADS v2[28].

To address class imbalance during training, we explored two approaches: focal loss and class weighting. The focal loss function was implemented to emphasise hard-to-classify samples by modulating the loss contribution based on prediction confidence. This technique introduces a focusing parameter that reduces the loss for well-classified examples and increases it for misclassified ones, particularly useful in imbalanced class scenarios. In parallel, class weights were computed and applied to the standard cross-entropy loss to penalise errors in minority classes more strongly. The weight for each class *i* was computed as $w_i = \frac{N}{k \times N_i}$, where *N* is the total number of pixels, $N_i$ is the number of pixels in class *i*, and *k* is the number of classes.

An ablation study was performed on the MultiResUNet model under the end-to-end two-stage architecture using three configurations: (1) standard cross-entropy loss, (2) cross-entropy with class weighting, and (3) focal loss. The goal was to empirically isolate the effect of each loss configuration on segmentation performance. The results were as follows:

- *Standard Cross-Entropy* Achieved the highest performance with a DSC of 0.804, HD of 4.82 mm, and ASD of 4.24 mm.
- *Class-Weighted Cross-Entropy* Performed moderately, with DSC = 0.612, HD = 8.05 mm, and ASD = 0.00 mm. The ASD value reflects prediction collapse during post-processing.
- *Focal Loss* Significantly underperformed, yielding DSC = 0.324, HD = 31.74 mm, and ASD = 0.64 mm.

This degradation can be attributed to several factors. First, although focal loss was implemented using its canonical formulation [34], with $\gamma =2$ and $\alpha =0.25$, these parameters were not systematically tuned for the prostate MRI segmentation task. Such defaults may not generalse optimally to fine-grained anatomical delineation [35]. Second, the dataset did not present severe class imbalance, which reduces the relative benefit of hard example mining [36]. Third, the end-to-end two-stage design already narrows inference to prostate-containing regions, mitigating the need for loss re-weighting mechanisms [20, 37]. Finally, the observed instability in the class-weighted variant. Specifically, performance collapse on certain metrics may be attributable to vanishing gradients of majority-class predictions [38]. Based on these outcomes, standard cross-entropy loss was selected for final deployment, offering the most stable and clinically interpretable performance.

**Fig. 10:**
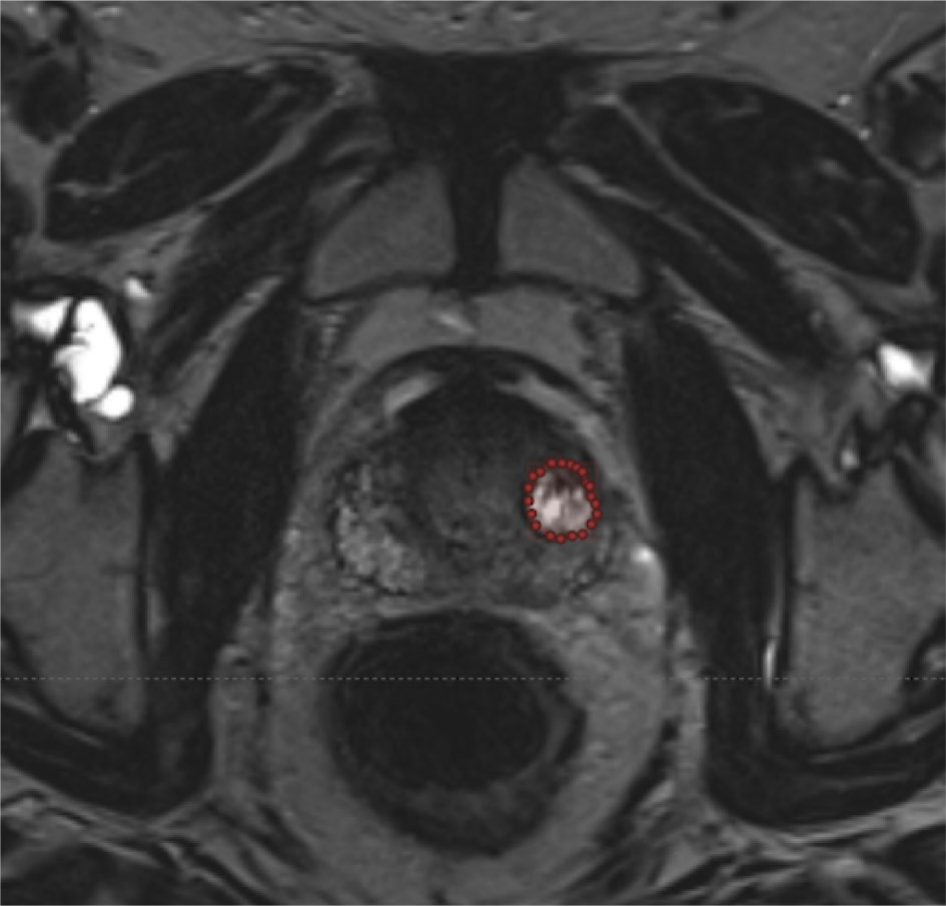
Axial t2-weighted MR image showing subtle low-signal-intensity area in anterior peripheral zone. Source: [28]

Some patients have been evaluated multiple times in the data set, but at different times, and can then show a change in the scoring. A characteristic of the data is that most patients are evaluated with a PI-RADS score of 3 (intermediate), 4 (high), or 5 (very high), showing a bias towards positive cancer cases. For example, Fig. 10 shows a t2-weighted MR image that hints at a tumour in the highlighted area. It is difficult to confirm a tumour from this image alone. However, using this evidence to support other techniques provides the possibility of an MRI-guided prostate biopsy that allows for more accurate targeting [39].

This paper uses an MR image dataset obtained from patients with biopsy-confirmed PCa. The annotations are stored in an annotation format with a link to the original DICOM file. The annotations are saved in a generalised format e.g. Table 1. The COCO (Common Objects in Context) format is a widely recognised standard for storing image data and annotations, especially in computer vision tasks. It provides a structured and flexible framework for linking annotations directly with their corresponding images, facilitating the organisation and retrieval of data for training machine learning models. In this paper’s approach, additional fields for Patient ID and an Expert Annotation flag have been included.

**Table 1:** Example of annotations saved in a generalised format and structure

Images	Patient	Annotations	Categories	ExpertAnn
Figure 11	0001	Figure 11	Figure 11	TRUE
Figure 11	0002	Figure 11	Figure 11	TRUE
Figure 11	0003	Figure 11	Figure 11	TRUE
Figure 11	–	Figure 11	Figure 11	FALSE
Figure 11	1151	Figure 11	Figure 11	FALSE

Data model for annotations includes references to original images, patient reference number, annotations location information, categorisation of annotation areas and the additional property of annotation method

**Fig. 11:**
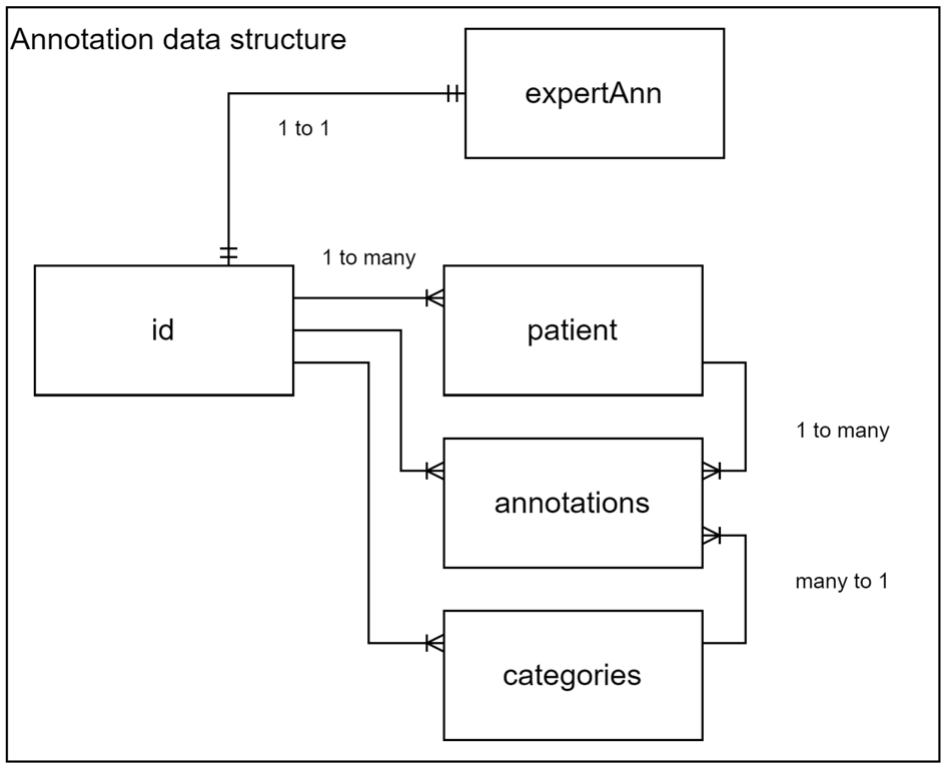
Properties of annotations presented in the diagram provides the references, relationships and categorisation to facilitate training activities. Additional details of annotations properties described in Table 1

## Results

The evaluation of the segmentation models is divided into three primary approaches: one-stage, two-stage sequential, and two-stage end-to-end. Each approach utilises multiple architectures, and the performance metrics are presented in Table 2. As seen in this table, the most notable improvement in Dice Similarity Coefficient (DSC) and Hausdorff Distance (HD) was observed with the MultiResUNet model in the two-stage end-to-end approach. This finding underscores the importance of leveraging multi-stage training to enhance segmentation accuracy in medical imaging.

**Table 2:** Summary of segmentation performance metrics (Dice similarity coefficient (DSC), Relative volume difference (RVD), Hausdorff distance (HD), Average surface distance (ASD), Combined specificity, Combined sensitivity, and Combined accuracy) across different model architectures and approaches (one-stage, two-stage sequential, and two-stage end-to-end). The table presents both the average and standard deviation values for each metric in the format “average ± standard deviation.” Additionally, class-specific metrics for the transition zone (TZ) and peripheral zone (PZ) are provided for each model. The MultiResUNet model in the two-stage end-to-end approach achieved the highest DSC (0.804) and the lowest HD (4.818), indicating increased performance in prostate segmentation compared to other models and approaches. These results underscore the effectiveness of multi-stage segmentation strategies in improving prostate MR image segmentation accuracy. Bold values indicate the best performance for each metric within a given approach.

Approach	Model architecture	DSC	RVD	HD	ASD	Specificity	Sensitivity	Accuracy
One-stage	Dense U-Net	0.318 ± 0.093	0.932 ± 0.034	28.244 ± 1.738	4.714 ± 4.048	0.793	0.325	0.779
	*TZ*	0.417 ± 0.028	0.932 ± 0.034	28.222 ± 1.745	4.793 ± 4.035	0.911	0.562	0.910
	*PZ*	0.357 ± 0.290	0.932 ± 0.034	28.222 ± 1.745	4.793 ± 4.035	0.924	0.179	0.917
	SegNet	0.428 ± 0.119	0.533 ± 0.251	**10.653 **±** 2.592**	4.706 ± 3.822	**0**.**879**	0.429	0.951
	*TZ*	0.547 ± 0.330	0.533 ± 0.251	10.612 ± 2.594	4.785 ± 3.805	0.952	0.556	0.951
	*PZ*	0.510 ± 0.374	0.533 ± 0.251	10.612 ± 2.594	4.785 ± 3.805	0.949	0.556	0.944
	Attention U-Net	0.392 ± 0.088	0.641 ± 0.200	13.000 ± 3.799	5.204 ± 4.253	0.868	0.408	0.908
	*TZ*	0.515 ± 0.327	0.642 ± 0.199	12.913 ± 3.770	5.291 ± 4.235	0.934	0.591	0.934
	*PZ*	0.432 ± 0.383	0.642 ± 0.199	12.913 ± 3.770	5.291 ± 4.235	0.924	0.174	0.917
	DeepLabV3	0.408 ± 0.099	0.608 ± 0.199	12.094 ± 2.189	5.022 ± 4.061	0.875	0.408	0.942
	*TZ*	0.530 ± 0.338	0.608 ± 0.199	12.058 ± 2.189	5.106 ± 4.042	0.944	0.553	0.944
	*PZ*	0.468 ± 0.386	0.608 ± 0.199	12.058 ± 2.189	5.106 ± 4.042	0.941	0.185	0.935
	RAUnet	0.367 ± 0.078	0.687 ± 0.167	12.931 ± 2.370	6.062 ± 4.950	0.860	0.385	0.913
	*TZ*	0.476 ± 0.336	0.687 ± 0.167	12.892 ± 2.371	6.163 ± 4.928	0.904	0.595	0.904
	*PZ*	0.396 ± 0.396	0.687 ± 0.167	12.892 ± 2.371	6.163 ± 4.928	0.922	0.106	0.914
	MultiResUnet	0.387 ± 0.095	0.749 ± 0.131	15.270 ± 2.554	4.962 ± 4.034	0.861	0.389	0.916
	*TZ*	0.515 ± 0.318	0.749 ± 0.131	15.256 ± 2.573	5.045 ± 4.016	0.944	0.562	0.943
	*PZ*	0.424 ± 0.385	0.749 ± 0.131	15.256 ± 2.573	5.045 ± 4.016	0.928	0.151	0.922
	PSPNet	**0.435 **±** 0.120**	**0.592 **±** 0.204**	11.903 ± 2.180	**4.565 **±** 3.691**	0.878	**0**.**435**	**0**.**949**
	*TZ*	0.579 ± 0.312	0.592 ± 0.205	11.875 ± 2.187	4.642 ± 3.674	0.955	0.578	0.955
	*PZ*	0.500 ± 0.374	0.592 ± 0.205	11.875 ± 2.187	4.642 ± 3.674	0.946	0.255	0.942
Two-stage sequential	Dense U-Net	0.513 ± 0.112	0.480 ± 0.273	9.473 ± 2.215	**6.853 **±** 0.645**	0.603	0.338	0.592
	*TZ*	0.205 ± 0.207	0.434 ± 0.380	2.749 ± 2.275	2.149 ± 1.926	0.593	0.540	0.591
	*PZ*	0.147 ± 0.202	0.278 ± 0.325	2.856 ± 2.652	0.644 ± 0.765	0.591	0.232	0.585
	SegNet	**0.523 **±** 0.090**	**0.457 **±** 0.268**	9.513 ± 1.835	7.220 ± 0.679	**0**.**604**	**0**.**353**	**0**.**593**
	*TZ*	0.208 ± 0.205	0.462 ± 0.384	3.009 ± 2.435	2.494 ± 2.043	0.592	0.577	0.591
	*PZ*	0.161 ± 0.190	0.268 ± 0.312	2.749 ± 2.542	0.744 ± 0.734	0.595	0.253	0.589
	Attention U-Net	0.489 ± 0.096	0.542 ± 0.233	9.619 ± 2.094	7.463 ± 0.668	0.601	0.335	0.588
	*TZ*	0.208 ± 0.214	0.334 ± 0.321	5.932 ± 4.958	4.602 ± 3.666	0.589	0.598	0.589
	*PZ*	0.105 ± 0.150	0.334 ± 0.321	5.9320 ± 4.958	4.602 ± 3.666	0.586	0.174	0.579
	DeepLabV3+	0.515 ± 0.088	0.527 ± 0.240	9.545 ± 1.801	7.546 ± 0.493	**0**.**604**	0.344	0.591
	*TZ*	0.197 ± 0.193	0.440 ± 0.375	2.716 ± 2.204	2.065 ± 1.873	0.594	0.519	0.592
	*PZ*	0.160 ± 0.201	0.321 ± 0.331	2.812 ± 2.601	0.972 ± 1.000	0.588	0.281	0.583
	RAUnet	0.504 ± 0.101	0.539 ± 0.235	9.690 ± 2.279	7.681 ± 0.659	0.602	0.341	0.589
	*TZ*	0.197 ± 0.200	0.447 ± 0.383	2.939 ± 2.387	2.373 ± 2.148	0.591	0.540	0.590
	*PZ*	0.143 ± 0.183	0.324 ± 0.332	2.799 ± 2.630	0.774 ± 0.879	0.586	0.254	0.581
	MultiResUnet	0.506 ± 0.101	0.527 ± 0.244	9.786 ± 2.188	7.584 ± 0.705	0.586	0.335	0.573
	*TZ*	0.206 ± 0.215	0.447 ± 0.387	2.885 ± 2.414	2.569 ± 2.193	0.575	0.565	0.575
	*PZ*	0.128 ± 0.172	0.311 ± 0.331	2.758 ± 2.627	0.545 ± 0.630	0.570	0.220	0.564
	PSPNet	0.514 ± 0.089	0.462 ± 0.255	**8.791 **±** 2.005**	6.894 ± 0.493	0.587	0.328	0.577
	*TZ*	0.221 ± 0.233	0.431 ± 0.381	2.676 ± 2.241	2.578 ± 2.199	0.578	0.567	0.577
	*PZ*	0.122 ± 0.162	0.276 ± 0.316	2.585 ± 2.471	0.457 ± 0.536	0.576	0.179	0.570
Two-stage end-to-end	Dense U-Net	0.428 ± 0.464	0.601 ± 0.474	20.930 ± 16.502	47.106 ± 0.531	0.383	0.514	0.1.0
	*TZ*	0.383 ± 0.486	0.0 ± 0.0	N/A	N/A	0.617	0.000	0.608
	*PZ*	0.517 ± 0.499	0.0 ± 0.0	N/A	N/A	0.617	0.000	0.612
	SegNet	0.615 ± 0.141	0.655 ± 0.173	19.036 ± 11.961	3.047 ± 4.042	0.225	**0**.**799**	0.781
	*TZ*	0.335 ± 0.272	0.296 ± 0.312	3.128 ± 0.401	2.758 ± 1.213	0.994	0.542	0.993
	*PZ*	0.182 ± 0.324	0.332 ± 0.449	4.603 ± 0.834	0.622 ± 0.669	0.999	0.015	0.995
	Attention U-Net	0.724 ± 0.222	0.391 ± 0.325	7.230 ± 8.387	3.733 ± 1.842	0.530	0.636	0.943
	*TZ*	0.632 ± 0.347	0.225 ± 0.254	2.637 ± 0.625	1.909 ± 0.919	0.665	0.353	0.663
	*PZ*	0.515 ± 0.419	0.277 ± 0.312	3.573 ± 0.674	0.973 ± 0.711	0.664	0.208	0.661
	DeepLabV3+	0.705 ± 0.256	0.414 ± 0.387	6.684 ± 6.070	**0.391 **±** 0.325**	0.507	0.627	0.917
	*TZ*	0.605 ± 0.374	0.213 ± 0.257	3.319 ± 0.234	2.177 ± 0.933	0.647	0.298	0.644
	*PZ*	0.504 ± 0.496	0.105 ± 0.293	4.773 ± 0.470	0.122 ± 0.154	0.650	0.002	0.645
	RAUnet	0.747 ± 0.220	0.443 ± 0.364	7.386 ± 7.792	4.311 ± 2.767	0.555	0.617	0.957
	*TZ*	0.686 ± 0.303	0.194 ± 0.220	2.769 ± 0.575	2.241 ± 1.235	0.630	0.451	0.629
	*PZ*	0.570 ± 0.444	0.302 ± 0.403	4.607 ± 0.703	1.218 ± 0.492	0.633	0.089	0.629
	MultiResUnet	**0.804 **±** 0.181**	0.348 ± 0.282	**4.818 **±** 4.111**	4.243 ± 1.115	**0**.**626**	0.615	**0**.**989**
	*TZ*	0.721 ± 0.253	0.245 ± 0.262	2.732 ± 0.427	2.288 ± 1.168	0.613	0.589	0.613
	*PZ*	0.695 ± 0.355	0.276 ± 0.324	3.449 ± 0.747	1.509 ± 0.489	0.615	0.258	0.614
	PSPNet	0.779 ± 0.204	**0.316 **±** 0.305**	6.518 ± 7.823	4.190 ± 2.416	0.574	0.617	0.970
	*TZ*	0.655 ± 0.350	0.203 ± 0.255	2.880 ± 0.612	2.322 ± 0.923	0.630	0.397	0.628
	*PZ*	0.692 ± 0.339	0.166 ± 0.212	3.485 ± 0.581	1.335 ± 0.478	0.629	0.258	0.627

Bold values indicate the best performance for each metric within a given approach

### One-stage approach

The one-stage approach involves training the model in a straightforward manner, where the segmentation task is treated as a single problem. Figure 22 showcases the segmentation results across different slices using this approach. Although the model architectures, such as Attention U-Net and MultiResUNet, demonstrated relatively good performance, it became evident that the one-stage method struggles with capturing intricate prostate zone boundaries accurately. This is especially noticeable in the overlap regions between the Transition Zone (TZ) and Peripheral Zone (PZ).

The quantitative results from Table 2 show that the one-stage approach generally achieves lower DSC and higher HD, indicating less accurate segmentation. This could be attributed to the complexity of multi-class segmentation tasks, where simultaneous learning of all classes in a single step is inherently more challenging. Consequently, this prompted to explore the two-stage approaches to improve segmentation accuracy.

### Two-stage sequential approach

In the two-stage sequential approach, the segmentation task is broken into two distinct phases: first, identifying the presence of the prostate and then performing the segmentation of the specific zones. Figure 23 demonstrates the improved segmentation results using this method. It is evident that segmenting in two stages allows the models to focus more precisely on the prostate regions, leading to better-defined boundaries and improved overlap with the ground truth.

Comparing the results between the one-stage and two-stage sequential approaches, we observe an overall increase in segmentation accuracy, as indicated by improved DSC and reduced HD. This improvement validates the hypothesis that dividing the task into smaller, more manageable steps facilitates more accurate learning by the model. Notably, architectures like DeepLabV3+ and RAUNet excelled in this setup, as reflected by their consistently high DSC scores in Table 2.

### Two-stage end-to-end approach

The two-stage end-to-end approach integrates the prostate presence detection and segmentation tasks in a more cohesive manner, enabling the model to learn these tasks concurrently. The results shown in Fig. 24 clearly illustrate that this approach outperforms both the one-stage and sequential methods. The MultiResUNet, in particular, achieved the highest DSC and lowest HD, as indicated in Table 2, demonstrating its ability to capture the fine-grained details of the prostate zones.

The performance of the two-stage end-to-end approach can be attributed to the model’s ability to leverage contextual information from both stages simultaneously, resulting in more accurate segmentation boundaries, as indicated in Table 2 and demonstrates the potential of using end-to-end learning frameworks for complex medical image segmentation tasks.

**Table 3:** *P*-values from two-sided t-tests comparing top-performing models (MultiResUNet, PSPNet, RAUnet) under the two-stage end-to-end configuration across three key metrics: Dice similarity coefficient (DSC), Hausdorff distance (HD), and Average surface distance (ASD)

Comparison	DSC (p)	HD (p)	ASD (p)
MultiResUNet vs PSPNet	0.508	0.052	0.821
MultiResUNet vs RAUnet	0.674	0.859	0.164
PSPNet vs RAUnet	0.545	0.732	0.370

To assess whether performance differences between models were statistically significant, we performed two-sided t-tests comparing the top three models—MultiResUNet, PSPNet, and RAUnet—across three key segmentation metrics: Dice Similarity Coefficient (DSC), Hausdorff Distance (HD), and Average Surface Distance (ASD). The results, presented in Table 3, indicate that although MultiResUNet achieved the highest average DSC (0.804) and lowest HD (4.818 mm), the observed improvements were not statistically significant compared to PSPNet and RAUnet (e.g., p = 0.508 for DSC between MultiResUNet and PSPNet). This underscores the importance of considering both mean performance and variability in model evaluation.

### Evaluation of accuracy, sensitivity, and specificity

As shown in Table 2, the MultiResUNet in the two-stage end-to-end approach achieved the highest overall accuracy (0.989), indicating a strong ability to predict the correct segmentation labels. However, while accuracy is high, the specificity (0.626) and sensitivity (0.615) reflect a nuanced challenge in differentiating true positives from false positives. The relatively balanced sensitivity and specificity suggest that the model is effective at identifying prostate zones while avoiding over-predictions, but still has room for improvement in precisely delineating prostate boundaries, particularly in anatomically complex regions such as the Transition Zone (TZ) and Peripheral Zone (PZ).

**Fig. 12:**
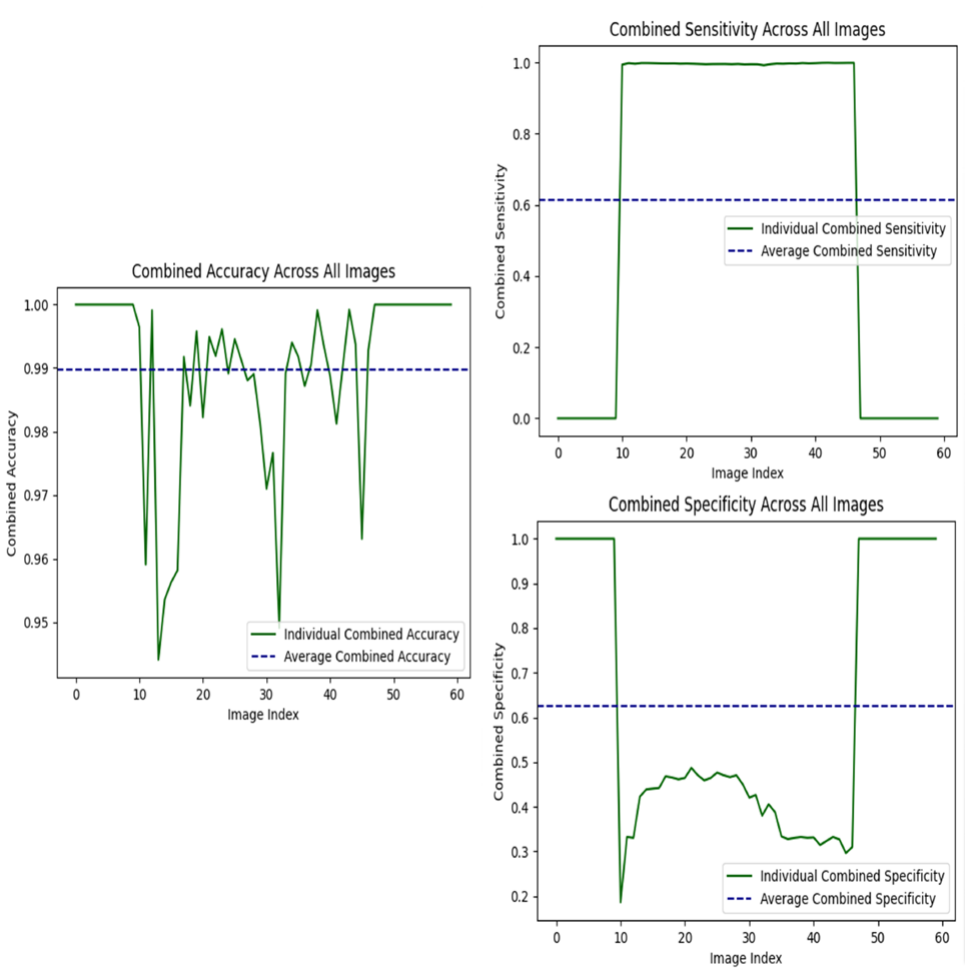
Visualisations of combined metrics (accuracy, specificity, sensitivity) across all images in a two-stage end-to-end approach using the MultiResUNet: the left plot represents the combined accuracy, showing the individual accuracy values for each image in green and the overall average accuracy as a dark blue dashed line. The top-right plot illustrates the combined sensitivity across all images, highlighting variations between individual sensitivities (green line) and the averaged sensitivity (dark blue dashed line). Similarly, the bottom-right plot showcases the combined specificity, with the individual and average values depicted in green and dark blue, respectively. These visualisations emphasise the model’s ability to consistently achieve high performance metrics, with minimal variation across images, while also providing a clear understanding of how individual metrics contribute to the overall average

The combined accuracy, specificity, and sensitivity metrics are visualised in Fig. 12, showing individual metric variations across images alongside their respective averages. The figure highlights that while the overall performance metrics are high, there are fluctuations in individual cases, particularly for sensitivity and specificity. These variations indicate that while the model performs well on average, certain images may pose challenges due to complexities in prostate anatomy or noise in the data.

The high accuracy, paired with the slightly lower sensitivity and specificity, suggests that the model correctly labels the majority of cases but may still struggle with minority classes or boundary precision. This may reflect a potential class imbalance or dependency between the binary classification and segmentation stages, where errors in one stage propagate to the next, impacting overall sensitivity and specificity. For comparison, the SegNet model in the same two-stage end-to-end approach demonstrated higher sensitivity (0.799) but significantly lower specificity (0.225), indicating a tendency to over-predict prostate regions. This high sensitivity is advantageous for detecting any prostate tissue but may lead to an increased number of false positives, compromising segmentation reliability.

These results emphasise the importance of balancing sensitivity and specificity to achieve optimal segmentation performance. The visualisations in Fig. 12 further confirm that while high accuracy is a strong indicator of overall performance, sensitivity and specificity provide deeper insights into how well the model handles the finer details of prostate segmentation, particularly in distinguishing between different prostate zones and background regions.

The high accuracy in the binary classification (prostate presence detection) is evident from the consistently high true positive and true negative rates, as visualised in Fig. 12. However, in the segmentation task, a more nuanced interpretation is required due to the complexity of distinguishing between multiple prostate zones (TZ and PZ). The Transition Zone (TZ) segmentation demonstrates some false positives and false negatives, indicating occasional misclassification of adjacent zones or background as TZ. This challenge likely arises from the anatomical proximity and overlapping boundaries of the prostate zones, which are common issues in medical imaging of complex regions.

Similarly, the Peripheral Zone (PZ) segmentation reveals periodic misclassification, particularly due to the subtle and variable appearance of peripheral tissues. Overlapping textures between the PZ and TZ and variations in peripheral anatomy contribute to these errors. These challenges highlight the inherent difficulty of accurately segmenting smaller or less distinct regions, despite the overall high accuracy and robust performance of the model. The visualised metrics further emphasise the importance of balancing sensitivity and specificity in addressing these nuanced segmentation challenges.

### Training and validation analysis

**Fig. 13:**
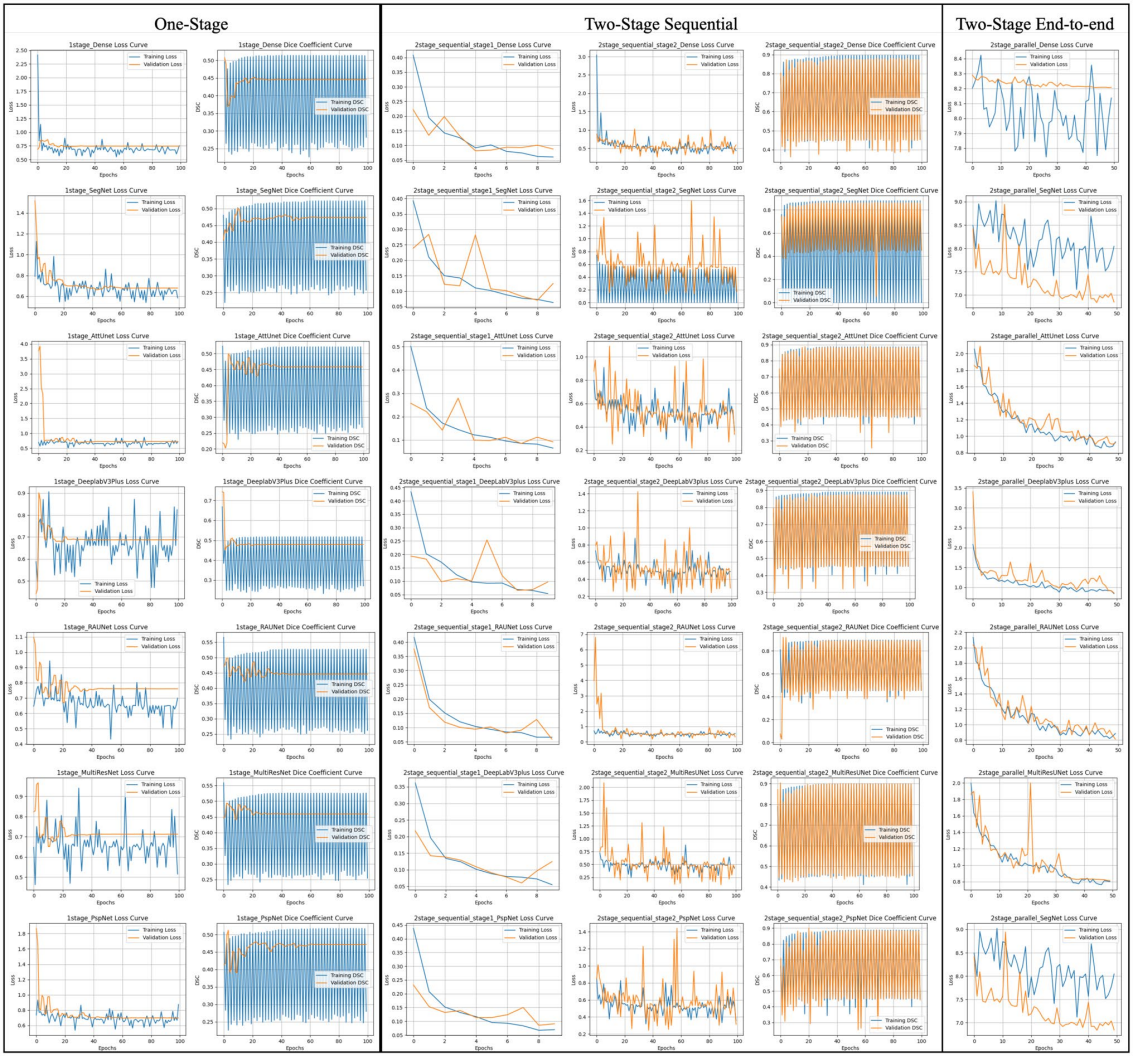
Training and validation metrics across different model architectures and stage approaches: this figure presents the training and validation loss curves (left) and Dice Coefficient curves (right) for each model architecture (Dense U-Net, SegNet, Attention U-Net, DeepLabV3+, RAUNet, MultiResUNet, and PSPNet) across three different stage approaches: one-stage, two-stage sequential, and two-stage end-to-end. The loss and Dice Coefficient curves allow for a comparative analysis of the convergence behaviour and segmentation performance for each model. The blue lines represent the training phase, while the orange lines denote the validation phase. Each row corresponds to a specific model architecture, and each column represents the stage approach used, highlighting how different configurations influence model performance during training. This comparison provides insight into the stability, learning rate, and overall performance achieved with each combination of architecture and segmentation stage approach

To gain insights into the training behaviour of different architectures, Fig. 13 presents the training and validation loss curves and Dice Coefficient (DSC) curves across all approaches. The MultiResUNet architecture showed the most stable and consistent learning patterns, particularly in the two-stage end-to-end setup, which aligns with the observed quantitative improvements. In contrast, the other architectures, such as Dense U-Net and SegNet, exhibited higher variability and slower convergence, indicating that the two-stage end-to-end approach is better suited for complex segmentation tasks like prostate MR image segmentation. To further assess the deployment feasibility of the proposed pipeline, we measured training and inference time for the best-performing configuration (MultiResUNet in the two-stage end-to-end pipeline). Training over 50 epochs with a batch size of 8 took 13,921 s. The inference time was 0.196 s per image. These results suggest that the proposed method is suitable for integration into real-time or near-real-time clinical workflows.

### Distribution analysis of evaluation metrics

**Fig. 14:**
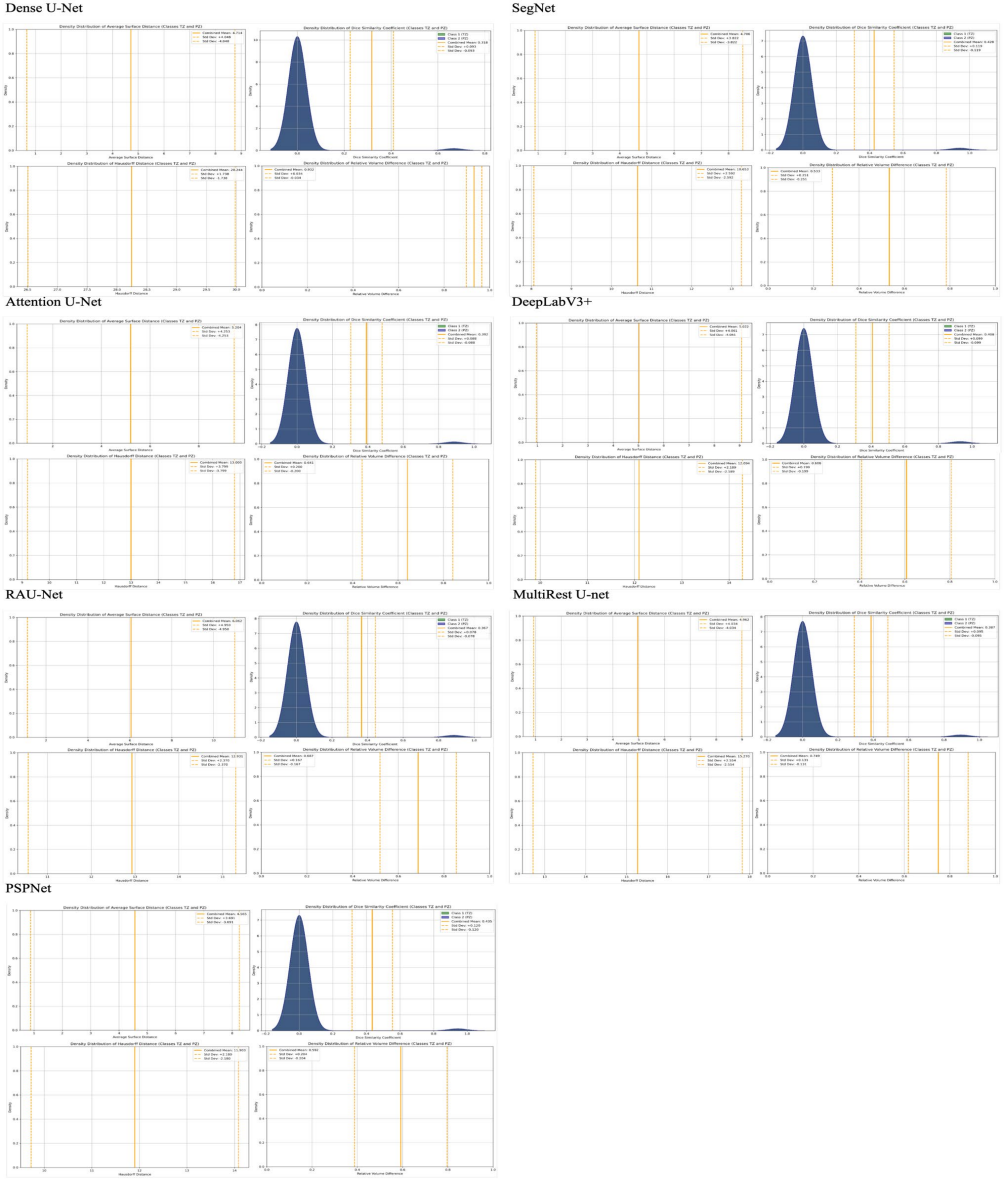
Density distribution of evaluation metrics across different model architectures for the one-stage approach: this figure presents the density distributions of two key evaluation metrics—Average Surface Distance (left) and Dice Similarity Coefficient (right)—for various model architectures (Dense U-Net, SegNet, Attention U-Net, DeepLabV3+, RAUNet, MultiResUNet, and PSPNet) under the one-stage segmentation approach. Each subplot displays the metric distributions per class: Class 1 (Transition Zone), Class 2 (Peripheral Zone), and the combined mean value, providing a comparative analysis of segmentation performance across different architectures. These distributions offer insight into the variability, accuracy, and consistency of each model’s segmentation capability. The dashed lines represent the mean and standard deviation for the combined classes, facilitating an evaluation of the overall performance

**Fig. 15:**
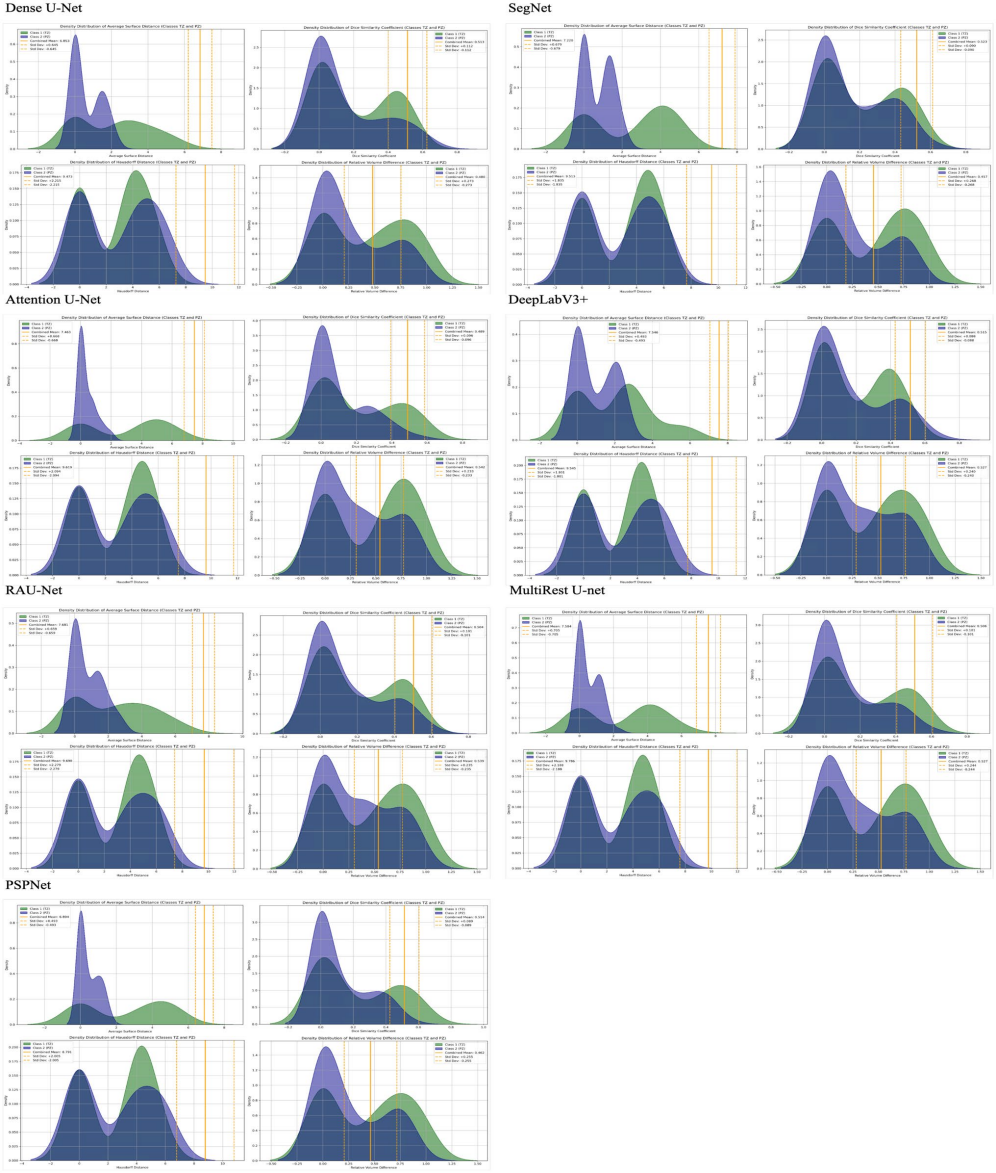
Density distribution of evaluation metrics across different model architectures for the two-stage sequential approach: this figure presents the density distributions of two key evaluation metrics—Average Surface Distance (left) and Dice Similarity Coefficient (right)—for various model architectures (Dense U-Net, SegNet, Attention U-Net, DeepLabV3+, RAUNet, MultiResUNet, and PSPNet) under the one-stage segmentation approach. Each subplot displays the metric distributions per class: Class 1 (Transition Zone), Class 2 (Peripheral Zone), and the combined mean value, providing a comparative analysis of segmentation performance across different architectures. These distributions offer insight into the variability, accuracy, and consistency of each model’s segmentation capability. The dashed lines represent the mean and standard deviation for the combined classes, facilitating an evaluation of the overall performance

**Fig. 16:**
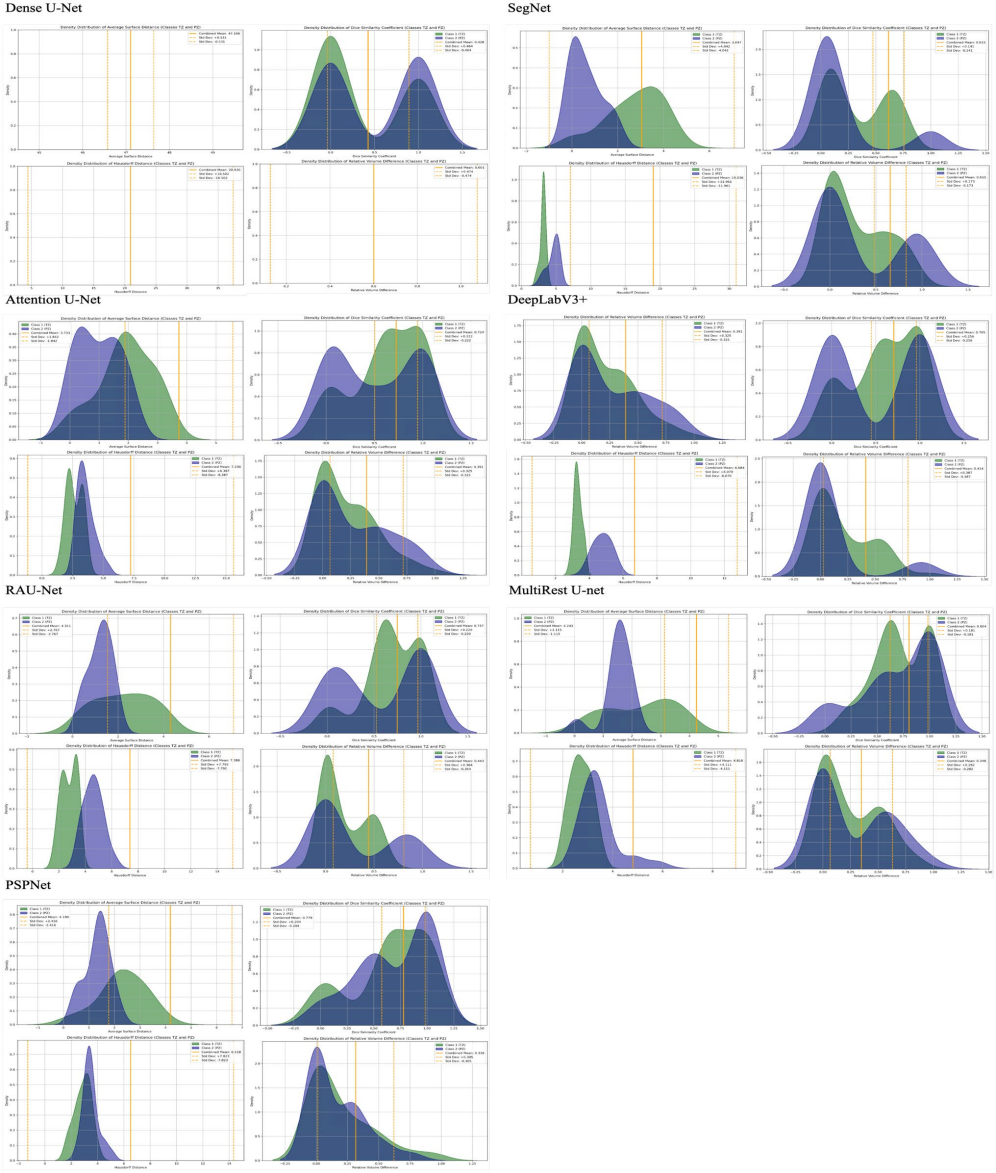
Density distribution of evaluation metrics across different model architectures for the two-stage end-to-end approach: this figure presents the density distributions of two key evaluation metrics—Average Surface Distance (left) and Dice Similarity Coefficient (right)—for various model architectures (Dense U-Net, SegNet, Attention U-Net, DeepLabV3+, RAUNet, MultiResUNet, and PSPNet) under the one-stage segmentation approach. Each subplot displays the metric distributions per class: Class 1 (Transition Zone), Class 2 (Peripheral Zone), and the combined mean value, providing a comparative analysis of segmentation performance across different architectures. These distributions offer insight into the variability, accuracy, and consistency of each model’s segmentation capability. The dashed lines represent the mean and standard deviation for the combined classes, facilitating an evaluation of the overall performance

Figures 14, 15 and 16 display the density distributions of key evaluation metrics across the one-stage, two-stage sequential, and two-stage end-to-end approaches, respectively. The overall trend demonstrates that the two-stage end-to-end approach yields the most concentrated and consistent metric distributions, particularly for the MultiResUNet architecture. This consistency is indicative of the model’s robustness in handling the variability inherent in medical imaging data, especially for TZ and PZ segmentation.

The distribution of the evaluation metrics is more balanced in the two-stage end-to-end approach. It suggests that segmenting the prostate in a holistic manner while concurrently learning the subtasks results in improved segmentation accuracy and reliability compared to the other evaluated approaches.

### Precision-recall curves analysis

**Fig. 17:**
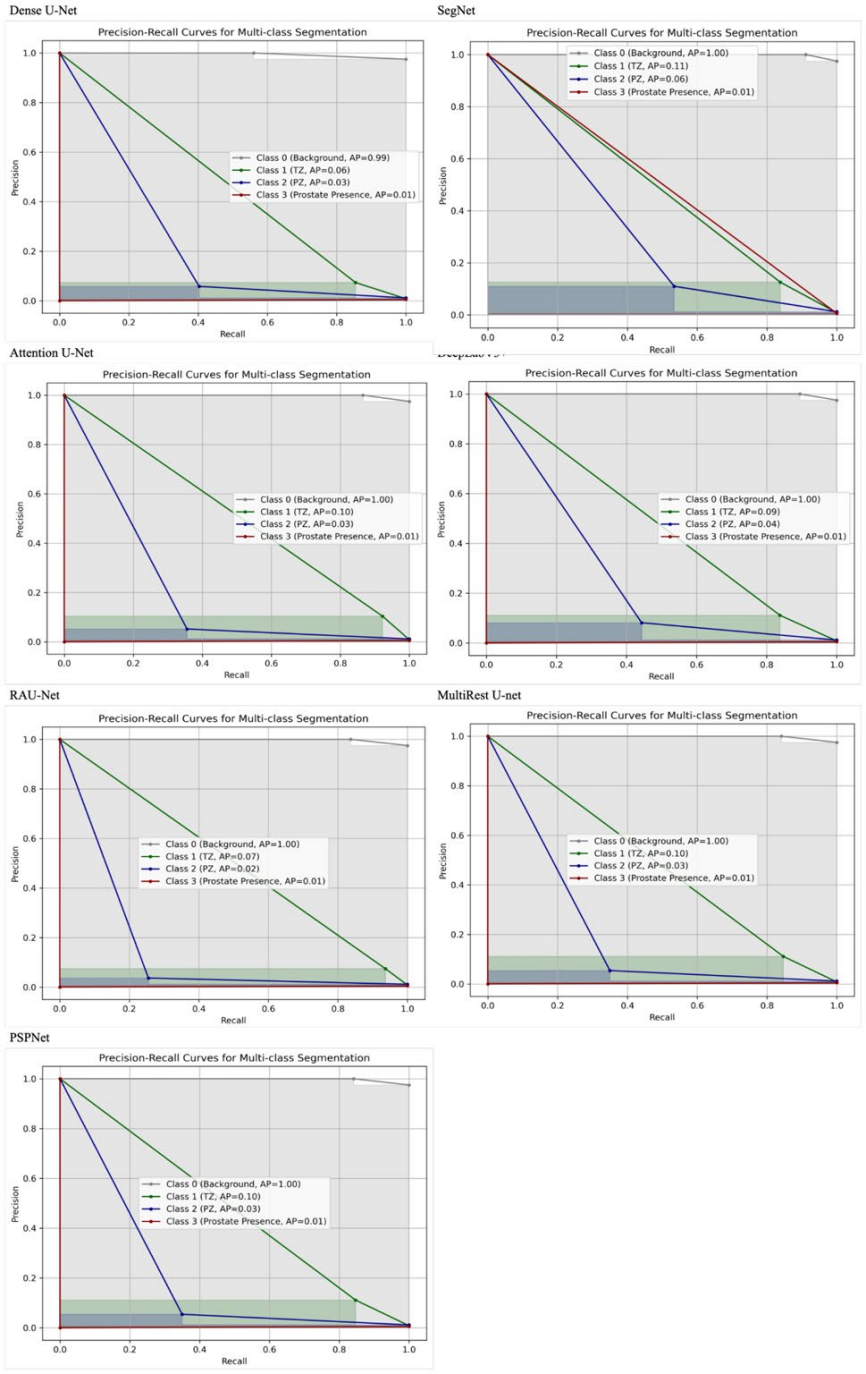
Precision-recall curves for the one-stage approach across different model architectures: this figure illustrates the precision-recall curves for various model architectures, namely Dense U-Net, SegNet, Attention U-Net, DeepLabV3+, RAUNet, MultiRes U-Net, and PSPNet, evaluated on the one-stage segmentation approach. The classes represented include Class 0 (Background), Class 1 (Transition Zone), Class 2 (Peripheral Zone), and Class 3 (Prostate Presence). Each curve includes the average precision (AP) values for the respective classes, providing a comparative analysis of how effectively each architecture handles multi-class segmentation within the end-to-end configuration. These curves highlight the models’ ability to differentiate between the prostate regions, offering insight into their performance within this specific segmentation approach

**Fig. 18:**
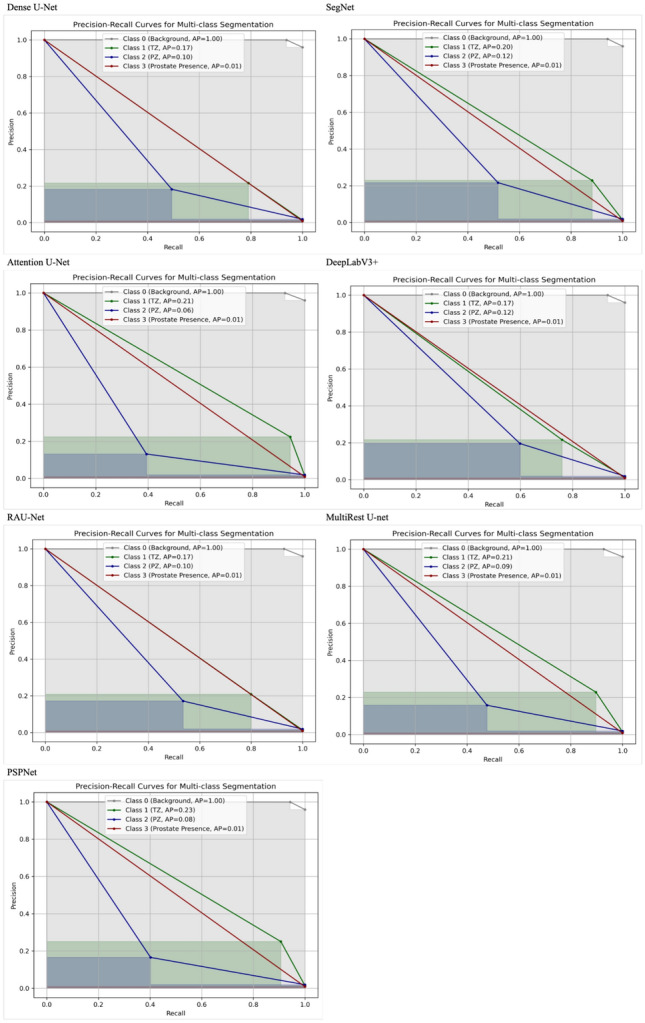
Precision-recall curves for the two-stage sequential approach across different model architectures: this figure illustrates the precision-recall curves for various model architectures, namely Dense U-Net, SegNet, Attention U-Net, DeepLabV3+, RAUNet, MultiRes U-Net, and PSPNet, evaluated on the two-stage sequential segmentation approach. The classes represented include Class 0 (Background), Class 1 (Transition Zone), Class 2 (Peripheral Zone), and Class 3 (Prostate Presence). Each curve includes the average precision (AP) values for the respective classes, providing a comparative analysis of how effectively each architecture handles multi-class segmentation within the end-to-end configuration. These curves highlight the models’ ability to differentiate between the prostate regions, offering insight into their performance within this specific segmentation approach

**Fig. 19:**
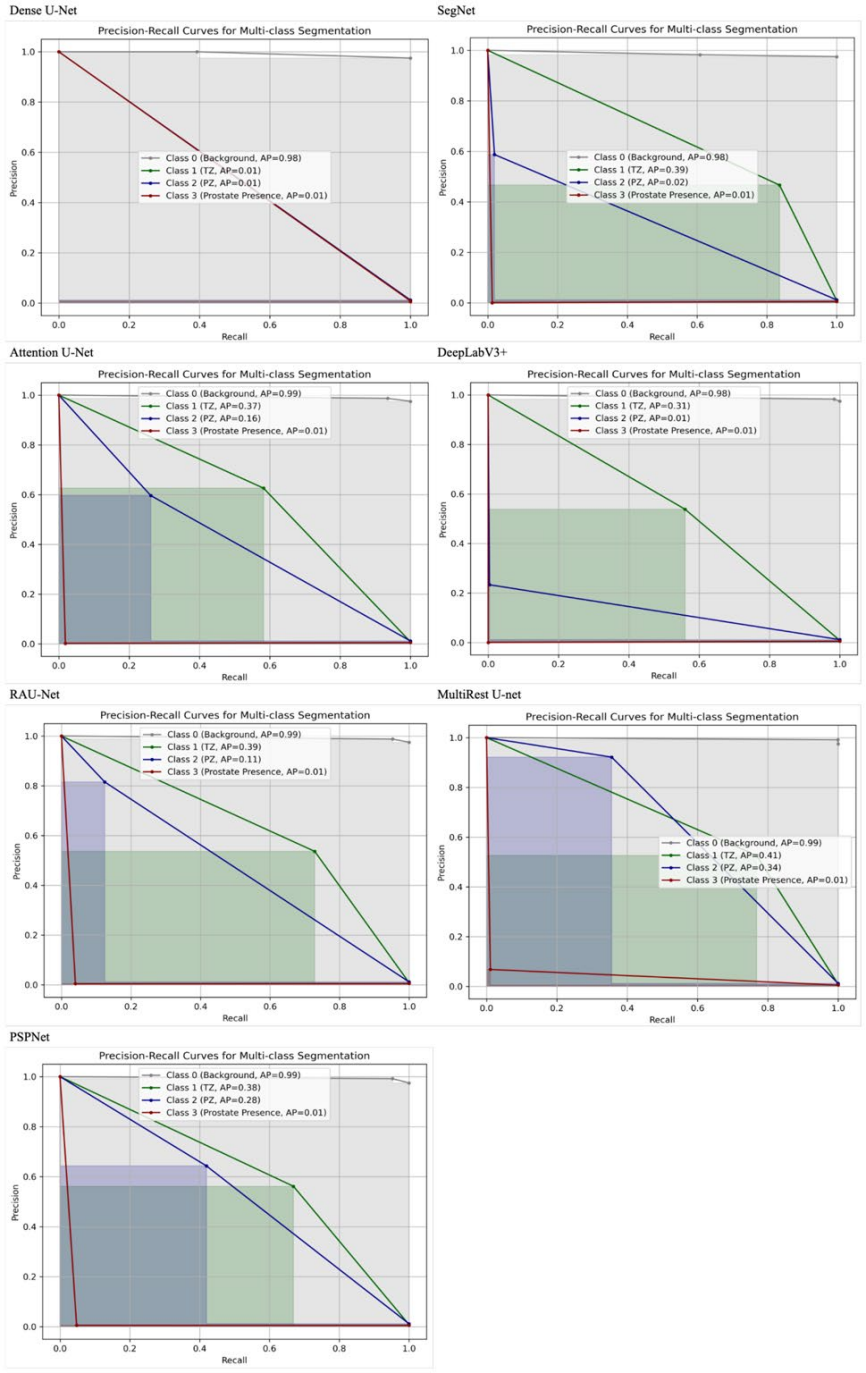
Precision-recall curves for the two-stage end-to-end approach across different model architectures: this figure illustrates the precision-recall curves for various model architectures, namely Dense U-Net, SegNet, Attention U-Net, DeepLabV3+, RAUNet, MultiRes U-Net, and PSPNet, evaluated on the two-stage end-to-end segmentation approach. The classes represented include Class 0 (Background), Class 1 (Transition Zone), Class 2 (Peripheral Zone), and Class 3 (Prostate Presence). Each curve includes the average precision (AP) values for the respective classes, providing a comparative analysis of how effectively each architecture handles multi-class segmentation within the end-to-end configuration. These curves highlight the models’ ability to differentiate between the prostate regions, offering insight into their performance within this specific segmentation approach

The precision-recall curves in Figs. 17, 18 and 19 provide additional insights into the segmentation performance across different architectures and approaches. The MultiResUNet model consistently achieved higher precision and recall values, particularly for Class 1 (TZ) and Class 2 (PZ), in the two-stage end-to-end setup. This indicates that the model effectively captures true positive regions while minimising false positives, which is crucial for clinical applications where accurate segmentation is essential.

### External validation performance

To evaluate the generalisability of the proposed segmentation pipeline, we applied the best-performing model—*MultiResUNet within the Two-Stage End-to-End framework*—to an independent subset of 100 patients from the publicly available PI-CAI dataset [29], using the zonal anatomical annotations provided by Yuan et al. [30].

The model was applied without any retraining or fine-tuning. Evaluation was conducted using the same metrics as in the main study: Dice Similarity Coefficient (DSC) and Hausdorff Distance (HD). Due to discrepancies in mask formatting and structure in the PI-CAI annotations, Relative Volume Difference (RVD) and Average Surface Distance (ASD) could not be computed reliably and are excluded from this analysis (Table 4).

**Table 4:** Comparison of internal test results vs. external validation (PI-CAI) for the best-performing model

Metric	Internal test set	External validation (PI-CAI)
Mean DSC	$\mathbf {0.804 \pm 0.181}$	$0.729 \pm 0.325$
Mean HD (mm)	$4.818 \pm 4.111$	$3.854 \pm 4.640$
RVD	$0.348 \pm 0.282$	Not computed
ASD (mm)	$4.243 \pm 1.115$	Not computed

On the PI-CAI external dataset, the model achieved a mean Dice Similarity Coefficient (DSC) of 0.729 and a mean Hausdorff Distance (HD) of 3.85 mm. Compared to the internal test set performance (DSC = 0.804, HD = 4.82 mm), the DSC showed a moderate decline, while the HD improved slightly. These results indicate that the model retains a comparable level of performance when applied to an independent dataset with differing acquisition parameters and annotation protocols.

### Visual assessment: 3D representation

**Fig. 20:**
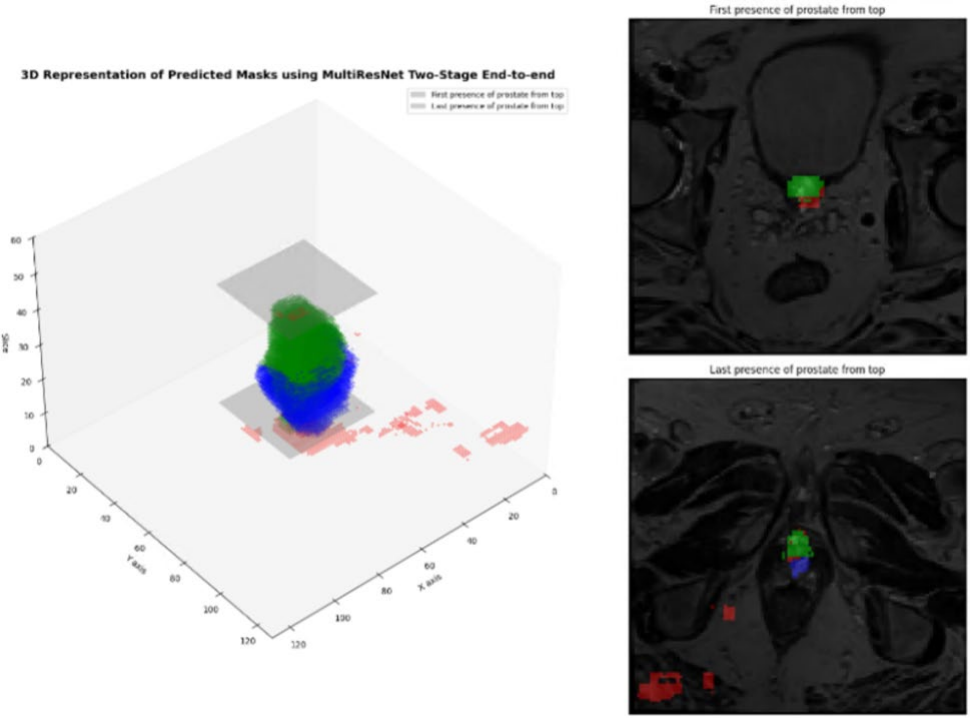
3D visualisation and slice representation of predicted prostate masks using the MultiResNet in the two-stage end-to-end approach: the left panel displays a 3D representation of the predicted prostate masks, highlighting the segmentation of different prostate zones—green for the Transition Zone (TZ), blue for the Peripheral Zone (PZ), and red for prostate presence traces. The right panel shows two specific slices from the original T2-weighted MR images, indicating the first (top) and last (bottom) presence of the prostate from the top view. These visualisations provide an insight into the segmentation accuracy achieved by the MultiResNet model in capturing the prostate structure and its zones in a multi-class end-to-end approach

Visualised in Fig. 20, a 3D representation of the predicted prostate masks using the MultiResNet model in the two-stage end-to-end approach. The visualisation shows the model’s ability to distinguish between the Transition Zone (TZ), Peripheral Zone (PZ), and prostate presence traces, confirming the effectiveness of the proposed approach. Notably, the figure visualises the inherent difficulty for the model to predict the beginning and the end of the anatomical prostate, as indicated by the gray square areas. This challenge is not only present in automated segmentation models but is also well recognised among radiologists, who often face difficulties in consistently identifying the exact boundaries of the prostate, especially at the base and apex. Accurate identification of these zones is crucial for clinical decision-making, as the transition between different anatomical zones can affect treatment planning, especially in cases of prostate cancer where the differentiation between TZ and PZ can significantly influence the detection of tumour lesions. This figure, therefore, underscores the potential of the model to assist radiologists by providing a detailed and precise delineation of prostate anatomy, thereby contributing to more accurate and comprehensive diagnosis and treatment planning in prostate cancer cases.

### Visual assessment: atypical prostate case

**Fig. 21:**
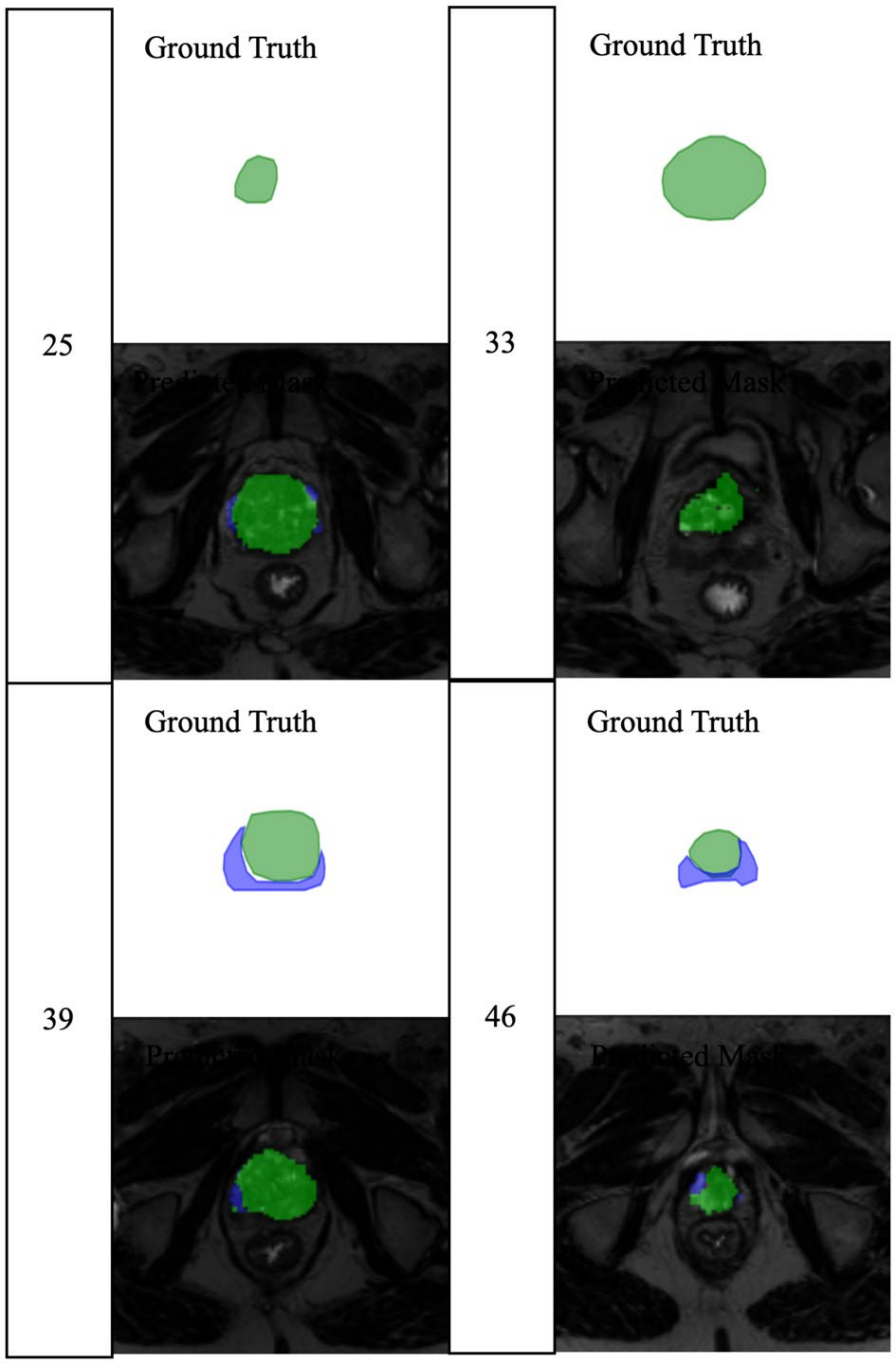
Ground truth vs predicted Masks for atypical prostate slices using the 2-stage parallel MultiResNet model: the figure showcases four selected slices (25, 33, 39, and 46) with the top row showing the ground truth segmentation and the bottom row showing the predicted masks. The model effectively captures the prostate shape in most regions, although discrepancies can be observed, particularly in the boundaries between the Transition Zone (TZ) and Peripheral Zone (PZ), underscoring the challenges in generalising to atypical prostate cases

**Fig. 22:**
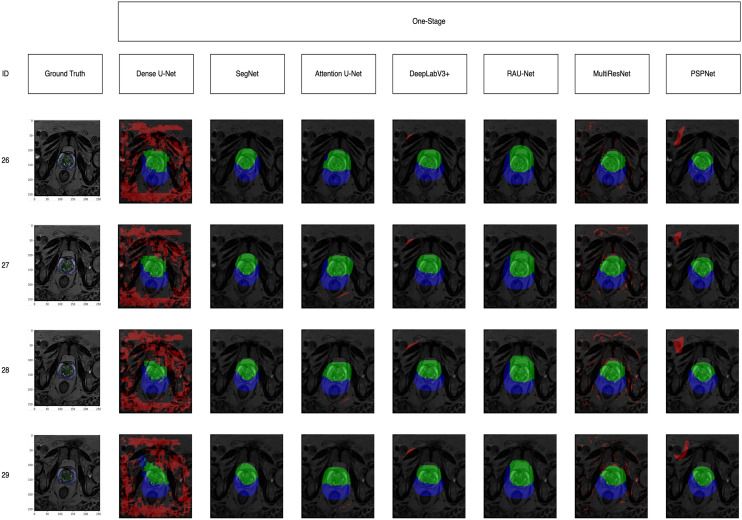
Results for the one-stage approach: This figure shows the segmentation results across different slices using the one-stage approach. The first row represents the ground truth, followed by the predicted segmentations using different architectures: Dense U-Net, SegNet, Attention U-Net, DeepLabV3+, RAUNet, MultiResUNet, and PSPNet. Each column represents different slices from the test data set

**Fig. 23:**
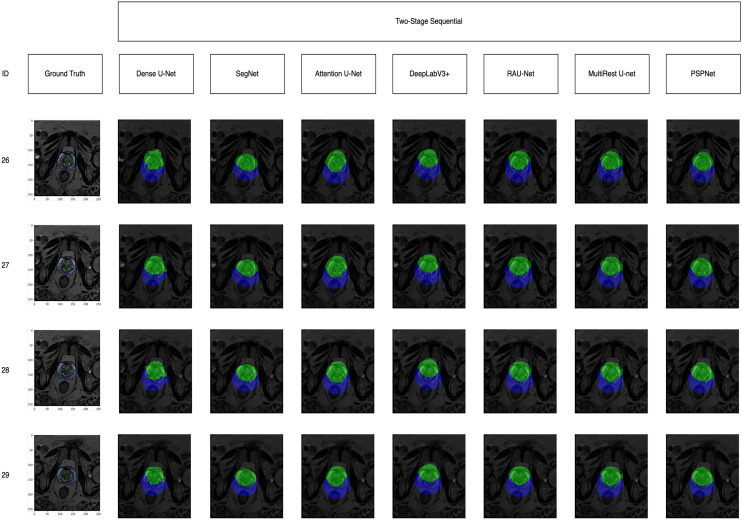
Results for the two-stage sequential approach: this figure shows the segmentation results across different slices using the two-stage sequential approach. The first row represents the ground truth, followed by the predicted segmentations using different architectures: Dense U-Net, SegNet, Attention U-Net, DeepLabV3+, RAUNet, MultiResUNet, and PSPNet. Each column represents different slices from the test data set

**Fig. 24:**
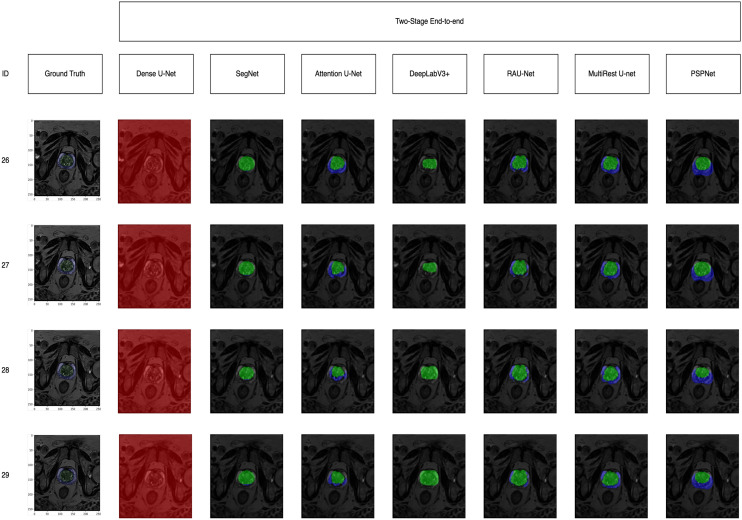
Results for the two-stage end-to-end approach: this figure shows the segmentation results across different slices using the two-stage end-to-end approach. The first row represents the ground truth, followed by the predicted segmentations using different architectures: Dense U-Net, SegNet, Attention U-Net, DeepLabV3+, RAUNet, MultiResUNet, and PSPNet. Each column represents different slices from the test data set

Figure 21 provides a comparison of ground truth and predicted Masks for an atypical prostate case, demonstrating the generalisability of the 2-stage parallel MultiResNet model. The four slices (25, 33, 39, and 46) show how the model handles different prostate shapes and complexities. The predicted masks (bottom row) show significant overlap with the ground truth (top row), but there are areas where discrepancies occur, particularly at the prostate boundaries. The variation between the Transition Zone (TZ) and the Peripheral Zone (PZ) boundaries poses additional segmentation challenges for this case.

Quantitative results for this challenging example yield a mean Dice Similarity Coefficient (DSC) of 0.761, which reflects a strong agreement between the predicted segmentation and ground truth. However, the relatively high standard deviation of 0.202 indicates variability across slices, suggesting that the model struggles to consistently capture prostate anatomy across the entire volume. Similarly, the mean Relative Volume Difference (RVD) of 0.3587 points to some inaccuracies in volume estimation, with a standard deviation of 0.3383, highlighting the model’s difficulty in maintaining volume consistency in this atypical case. Furthermore, the mean Hausdorff Distance (HD) of 10.79 mm, along with the high standard deviation of 11.34 mm, suggests substantial boundary deviations in certain slices, which is a critical factor in precise prostate delineation. The mean Average Surface Distance (ASD) of 2.96 mm, with a standard deviation of 2.69 mm, further emphasises the challenge of accurate surface approximation in more complex shapes, though overall surface distance remains relatively low. Overall underlining the model’s generalisability potential but also highlight the challenges of segmenting more complex prostate shapes, where the variability in anatomy and boundary definition are more pronounced Table 5.

**Table 5:** Evaluation metrics for the atypical prostate case using the 2-stage parallel multiResNet model

Metric	Mean	Standard deviation
Dice similarity coefficient (DSC)	0.761	0.202
Relative volume difference (RVD)	0.359	0.334
Hausdorff distance (HD) (mm)	10.79	11.34
Average surface distance (ASD) (mm)	2.96	2.69

### Comparative analysis with existing research

Several recent studies have reported Dice Similarity Coefficient (DSC) values ranging between 0.7 and 0.95 for prostate segmentation tasks, reflecting a high degree of spatial overlap between predicted and reference segmentations [14, 19, 20, 40]. Notably, Wang et al. achieved a DSC of 0.904 using a highly specialised network [14], while the nnUNet framework, considered a standardised baseline in medical image segmentation, reported a DSC of 0.746 for prostate segmentation [20]. In this context, the MultiResUNet model in this study achieved a DSC of 0.804±0.181 within the two-stage end-to-end pipeline. While slightly lower than the highest reported values, MultiResUNet’s performance remains competitive, especially considering variations in datasets, segmentation protocols, and model complexity. Importantly, the model achieved higher DSC compared to nnUNet, demonstrating that the proposed approach is well-aligned with or exceeds current performance benchmarks in the literature.

In terms of volume estimation, this study’s best-performing RVD (Relative Volume Difference) was 0.316±0.305, obtained using PSPNet within the two-stage end-to-end framework. This competitive values are reported by Tian et al., whose ConvLSTM and GGNN approach achieved an RVD of 0.024±0.126 [19]. While the numerical difference may suggest variability, it is important to note that many prior studies exclude non-prostate slices during evaluation, potentially inflating reported metrics. This study included full-volume slices, thus providing a more comprehensive assessment of model performance under clinical conditions.

When comparing boundary accuracy using the Hausdorff Distance (HD), the MultiResUNet model achieved a minimum HD of 4.818±4.111mm, lower than the nnUNet, which reported 6.046mm [20]. This reflects improved precision in delineating prostate contours. Furthermore, DeepLabV3+ achieved an average surface distance (ASD) of 0.391±0.325mm, which compares favourably against the 1.73mm reported by Tian et al. [19], indicating improved consistency and finer contour adherence. These findings confirm the effectiveness of the proposed two-stage design in improving boundary-level segmentation accuracy.

In contrast, the one-stage models explored in this study demonstrated Limited segmentation capability. For example, Dense U-Net and SegNet achieved DSCs of 0.318±0.093 and 0.428±0.119, respectively. Although these values are lower than those reported in the literature, they highlight the inherent limitations of single-stage pipelines in complex tasks like prostate segmentation. Other one-stage models such as PSPNet (DSC = 0.435±0.120) also fell short in comparison to the two-stage counterparts.

The two-stage sequential strategy offered moderate improvements. Dense U-Net, SegNet, and DeepLabV3+ recorded DSC values of 0.513±0.112, 0.523±0.090, and 0.515±0.088, respectively. These results align with findings from prior work suggesting that two-stage designs enhance feature extraction and spatial discrimination. Additionally, PSPNet achieved an RVD of 0.462±0.089 in this setting, reflecting more accurate volume estimations than in one-stage approaches.

The two-stage end-to-end strategy yielded the strongest results overall. In addition to the high DSC from MultiResUNet (0.804±0.181), other models such as RAUnet and PSPNet achieved DSCs of 0.747±0.220 and 0.779±0.204, respectively—values that are in line with or superior to those in comparable studies. MultiResUNet’s low HD and competitive ASD metrics further underscore its ability to capture fine-grained structural boundaries with high precision. Although the ASD reported for MultiResUNet was 4.243±1.115mm in one instance, this variation may be attributed to dataset characteristics and evaluation setup, as previous studies often perform selective slice analysis.

An important methodological distinction is that many previous works pre-process the MRI volumes by removing non-prostate-containing slices, thereby potentially inflating segmentation scores. In contrast, this study’s pipeline operates on full-volume data, preserving clinical realism and increasing generalisability.

In summary, while some studies report marginally higher segmentation scores, this study’s findings remain consistent with existing literature and demonstrate that this study’s proposed approach, particularly the two-stage end-to-end strategy with MultiResUNet, offers competitive and clinically meaningful performance. Furthermore, the relatively low architectural complexity of this study’s models, compared to more parameter-heavy alternatives, makes them viable for real-world deployment in resource-constrained settings.

## Conclusion

This study presents multiple approaches to prostate MR image segmentation, demonstrating the significant advantages of using a two-stage end-to-end methodology. The MultiResUNet model achieved the highest overall performance, with a Dice Similarity Coefficient (DSC) of 0.804 and a Hausdorff Distance (HD) of 4.818, indicating a high degree of accuracy in delineating prostate boundaries. These results highlight the model’s capacity to provide a reliable and robust solution for automated prostate segmentation tasks, which is crucial for improving prostate cancer diagnosis and treatment planning. In reviewing the performance across models, we observed that architectures such as SegNet achieved relatively high sensitivity but lower specificity. This imbalance can lead to over-segmentation, where non-prostate or surrounding tissues are incorrectly included in the segmented region. From a clinical perspective, such behaviour could inflate the apparent prostate volume or misrepresent tumour Margins, potentially influencing clinical decisions such as biopsy targeting or radiotherapy planning. To mitigate these risks, future implementations could incorporate anatomical shape priors, post-hoc filtering based on expected prostate volume ranges, or probabilistic uncertainty estimates to flag potentially erroneous segmentations. In contrast, the end-to-end two-stage MultiResUNet achieves a more balanced performance, indicating improved precision in delineating anatomical boundaries while minimising false positives and false negatives. This balance enhances its reliability and safety in real-world diagnostic workflows. External validation on the PI-CAI dataset further demonstrated that the proposed model retains generalisability across datasets, achieving a mean DSC of 0.729 and a mean HD of 3.85 mm without re-training, supporting its robustness under varying acquisition conditions and annotation standards.

Visual assessment (Fig. 20), offered critical insights into the model’s segmentation capabilities, particularly in distinguishing between the Transition Zone (TZ), Peripheral Zone (PZ), and overall prostate presence. This visualisation also underscores the model’s ability to tackle one of the more complex challenges in prostate segmentation: identifying the precise anatomical beginning and end of the prostate structure, a task that even experienced radiologists find challenging.

The comparative analysis with existing literature further supports the efficacy of the proposed methodology, showcasing improvements across several evaluation metrics, including DSC, RVD, and ASD. This underscores the potential of combining deep learning with domain-specific knowledge to enhance the accuracy and reliability of prostate segmentation models.

Moreover, the two-stage end-to-end approach demonstrated clear advantages over the one-stage and sequential methods in terms of segmentation precision and robustness, as evidenced by the comprehensive evaluation metrics. This finding suggests that integrating multiple segmentation stages allows for capturing more complex anatomical features, which is essential in medical imaging tasks where high accuracy is required.

The contribution of this paper extends beyond the development of a single model. It introduces a system that effectively integrates domain expertise and deep learning techniques, creating a framework that can be adapted and refined for broader applications in medical imaging. By leveraging a combination of quantitative and qualitative assessments, this research bridges the gap between automated segmentation and expert knowledge, offering a scalable solution that can be applied to enhance clinical decision-making in prostate cancer care.

Future work will address current limitations, including segmentation inaccuracies at the prostate base and apex, regions prone to weak boundary definition and high anatomical variability. To mitigate these challenges, future directions include integrating anatomical priors such as statistical shape models or atlas-based constraints, and exploring shape-aware or region-adaptive loss functions that can better guide learning in these complex zones. Attention-based mechanisms may further enhance context-sensitive segmentation in these anatomically variable regions. Although focal loss and class weighting are commonly used to address class imbalance in medical image segmentation, this ablation study revealed that neither approach improved model performance in this task.

Another key area for development is improving generalisation to unseen data. While the proposed model demonstrates strong performance on the TCIA dataset, domain shifts introduced by different scanners, acquisition protocols, or patient populations can limit clinical robustness. Future research will focus on domain adaptation strategies, such as adversarial training or feature normalisation, and emphasise external validation using multi-institutional datasets to ensure real-world applicability. Establishing cross-center collaborations will be crucial to evaluating the model’s transferability and minimising overfitting to dataset-specific characteristics.

This study also did not include transformer-based or hybrid CNN-transformer models, which are increasingly prominent in medical imaging. Future work should extend the evaluation framework to include such architectures, examining whether the benefits of the proposed end-to-end two-stage pipeline persist across these more advanced paradigms.

In summary, the proposed method, particularly the end-to-end two-stage model leveraging MultiResUNet—demonstrates performance compared to conventional CNN architectures, achieving a Dice Similarity Coefficient of 0.804 and a Hausdorff Distance of 4.82 mm. These results position it favourably within the current state-of-the-art and highlight its potential for integration into AI-assisted clinical workflows for prostate MRI interpretation. Continued improvements through architectural innovation, clinical validation, and cross-domain generalization are necessary steps toward widespread deployment in precision radiology.

## Data Availability

No datasets were generated or analysed during the current study.

## Detailed architecture specifications

This appendix provides further architectural details of the models used in the experiments. Each model architecture, including Dense U-Net, SegNet, Attention U-Net, DeepLabV3+, RAUNet, MultiResUNet, and PSPNet, has its specific configuration, as outlined below.

### Dense U-Net

The Dense U-Net consists of densely connected convolutional layers that improve information flow between layers. The architecture includes 5 dense blocks, each having 4 layers. The growth rate is set to 32, and dropout is applied after each dense block. The input is resized to 128x128 pixels with 3 channels (RGB) (table 6).

**Table 6:** Dense U-Net architecture summary

Layer	Parameters
Input layer	128 x 128 x 3
Conv2D	32 filters, kernel size 3 x 3
Dense block 1	4 layers, growth rate 32
Transition layer 1	Batch Normalisation, Pooling
Dense block 2	4 layers, growth rate 32
Transition layer 2	Batch Normalisation, Pooling
Dense block 3	4 layers, growth rate 32
Output layer	Softmax activation

### SegNet

SegNet is an encoder-decoder architecture. The encoder uses convolutional layers followed by max-pooling, and the decoder uses upsampling layers to reconstruct the spatial resolution. The model is optimised for semantic segmentation (Table 7).

**Table 7:** SegNet architecture summary

Layer	Parameters
Input layer	128 x 128 x 3
Conv2D	64 filters, kernel size 3 x 3
Max pooling	Pool size 2x2
Conv2D (Encoder)	128 filters, kernel size 3 x 3
UpSampling (Decoder)	Pool size 2 x 2
Output layer	Softmax activation

### Attention U-Net

Attention U-Net introduces attention gates in the encoder-decoder structure. These gates focus on relevant spatial features and filter irrelevant information to improve segmentation accuracy. The model employs skip connections between the encoder and decoder (Table 8).

**Table 8:** Attention U-Net architecture summary

Layer	Parameters
Input layer	128 x 128 x 3
Conv2D	64 filters, kernel size 3 x 3
Attention gate	Applied at skip connections
Max pooling	Pool size 2 x 2
UpSampling	Pool size 2 x 2
Output layer	Softmax activation

### DeepLabV3+

DeepLabV3+ is based on an encoder-decoder structure with atrous spatial pyramid pooling (ASPP). It captures multi-scale context by using dilated convolutions with different rates (Table 9).

**Table 9:** DeepLabV3+ architecture summary

Layer	Parameters
Input layer	128 x 128 x 3
Conv2D	64 filters, kernel size 3 x 3
ASPP	Dilation rates: 6, 12, 18
Decoder	Upsampling to restore spatial resolution
Output layer	Softmax activation

### RAUNet (residual attention U-Net)

RAUNet integrates residual connections and attention gates, enhancing the ability to focus on relevant regions within an image. The architecture includes residual blocks with attention gates applied in the encoder-decoder path (Table 10).

**Table 10:** RAUNet architecture summary

Layer	Parameters
Input layer	128 x 128 x 3
Conv2D	64 filters, kernel size 3 x 3
Residual block	Applied to every layer
Attention gate	Applied at skip connections
UpSampling	Pool size 2 x 2
Output layer	Softmax activation

### MultiResUNet

MultiResUNet uses multi-resolution blocks that incorporate convolutions with different kernel sizes (3x3, 5x5, and 7x7) to capture features at various scales. A residual path is added to each block for gradient flow. This model achieves enhanced segmentation performance across zones (Table 11).

**Table 11:** MultiResUNet architecture summary

Layer	Parameters
Input layer	128 x 128 x 3
MultiRes block 1	Filters: 32, Kernel sizes: 3 x 3, 5 x 5, 7 x 7
MultiRes block 2	Filters: 64, Kernel sizes: 3 x 3, 5 x 5, 7 x 7
Residual path	Applied between encoder and decoder
Output layer	Softmax activation

### PSPNet (pyramid scene parsing network)

PSPNet applies pyramid pooling to aggregate contextual information at different scales. The pyramid pooling module is crucial for capturing both local and global context (Table 12).

**Table 12:** PSPNet architecture summary

Layer	Parameters
Input layer	128 x 128 x 3
Conv2D	64 filters, kernel size 3 x 3
Pyramid pooling module	Bin sizes: 1, 2, 3, 6
Output layer	Softmax activation

## Data preprocessing steps

The following preprocessing steps were applied to all MR images as part of the training process:

1. **Normalisation**: All images were normalised to a range of [0, 1] using min-max scaling.
2. **Resizing**: Images were resized to 128x128 pixels to fit the input requirements of the models.
3. **colour Conversion**: If necessary, greyscale images were converted to RGB format.
4. **Augmentation**: Random rotations, translations, flips, and zooms were applied to increase the diversity of the training data.
5. **Class Label Mapping**: colour-coded masks were converted to class labels using tolerance-based mapping to ensure pixel-wise correspondence between classes.

Further details on preprocessing steps can be found in the example training script included in the main paper.

## Additional results

Additional segmentation results are presented in the following figures (Figs. 22, 23, 24).
